# Untargeted Metabolomics Profiling of an 80.5 km Simulated Treadmill Ultramarathon

**DOI:** 10.3390/metabo8010014

**Published:** 2018-02-13

**Authors:** Christopher C. F. Howe, Ahmed Alshehri, David Muggeridge, Alexander B. Mullen, Marie Boyd, Owen Spendiff, Hannah J. Moir, David G. Watson

**Affiliations:** 1 Applied & Human Sciences, School of Life Sciences, Pharmacy & Chemistry, Kingston University London, Kingston upon Thames KT1 2EE, UK; C.Howe@kingston.ac.uk (C.C.F.H.); O.Spendiff@kingston.ac.uk (O.S.); H.Moir@kingston.ac.uk (H.J.M.); 2 Strathclyde Institute of Pharmacy and Biomedical Sciences, University of Strathclyde, The John Arbuthnott Building, 161 Cathedral Street, Glasgow G4 0RE, UK; ahmed.alshehri@strath.ac.uk (A.A.); a.mullen@strath.ac.uk (A.B.M.); marie.boyd@strath.ac.uk (M.B.); 3 School of Psychological & Health Sciences, University of Strathclyde, Glasgow G4 0RE, UK; david.muggeridge@strath.ac.uk

**Keywords:** ultramarathon running, metabolomics, high resolution mass spectrometry, acyl carnitines, oxidized fatty acids

## Abstract

Metabolomic profiling of nine trained ultramarathon runners completing an 80.5 km self-paced treadmill-based time trial was carried out. Plasma samples were obtained from venous whole blood, collected at rest and on completion of the distance (post-80.5 km). The samples were analyzed by using high-resolution mass spectrometry in combination with both hydrophilic interaction (HILIC) and reversed phase (RP) chromatography. The extracted putatively identified features were modeled using Simca P 14.1 software (Umetrics, Umea, Sweden). A large number of amino acids decreased post-80.5 km and fatty acid metabolism was affected with an increase in the formation of medium-chain unsaturated and partially oxidized fatty acids and conjugates of fatty acids with carnitines. A possible explanation for the complex pattern of medium-chain and oxidized fatty acids formed is that the prolonged exercise provoked the proliferation of peroxisomes. The peroxisomes may provide a readily utilizable form of energy through formation of acetyl carnitine and other acyl carnitines for export to mitochondria in the muscles; and secondly may serve to regulate the levels of oxidized metabolites of long-chain fatty acids. This is the first study to provide evidence of the metabolic profile in response to prolonged ultramarathon running using an untargeted approach. The findings provide an insight to the effects of ultramarathon running on the metabolic specificities and alterations that may demonstrate cardio-protective effects.

## 1. Introduction

There has been an upward trend in life expectancy over the past few years in developed countries, but lifestyle risks still pose real challenges to longevity. These risks factors include obesity, unhealthy diet, cigarette smoking, sedentary lifestyle, and alcohol consumption [1]. Regular physical activity, coupled with a healthy balanced diet and moderate to no alcohol consumption, can significantly decrease the impact of these risk factors resulting in an increased life expectancy [2]. For instance, it has been reported that regular exercise attenuates sarcopenia and promotes cardiovascular health [3,4,5] as well as being prescribed for individuals with diabetes [6], obesity, and mild to moderate depression [5]. Moreover, it has been reported that incidences of hypertension, hypercholesterolemia, and diabetes decrease with the frequency of participation in marathons independent of the total distance run annually, but this might be due to longer training runs or genetic and innate differences between endurance trained marathon runners and those who are not [7]. It has been reported that compared to the general population ultra-endurance athletes have missed fewer work/school days through illness and injury and have overall lower incidences of chronic disease [8]. Although it has been suggested that sustained exercise such as ultra-marathon running may cause adverse cardiovascular effects [9], some studies suggest that chronic ultra-endurance training may attenuate biological ageing [10,11].

The rising popularity of ultramarathon running over the past few years has seen non-professional runners striving for bigger and tougher extreme physical challenges [8,12,13]. An ultramarathon is considered anything beyond a traditional marathon distance (42.2 km) with the majority of races being 80.5 km or more [12,14,15,16,17]. Of particular interest, is the increase in participation of runners with less than three years of regular running experience (25%) from the point of taking part in their first ultra-distance event [8]. Such extreme endurance events place a great deal of physiological and psychological demands on the body and with this increase in participation, and though it is perceived participants of such endurances, appear fit and healthy, there have been occasional reports of severe complications following ultra-endurance exercise and concern of harmful effects on health such as cardiac damage [9,18]. Ultramarathon runners place themselves at the risk of extreme fatigue, exhaustion and test their own personal limits [19]. Therefore, understanding the effects and implications of ultramarathon events is needed and the ultramarathon runners profile provides a unique model to investigate the physiological responses to prolonged physical exertion.

Ultra–endurance athletes push themselves beyond ordinary limits [20], with prolonged endurance exercise, eliciting extreme metabolic stress, inducing metabolic changes such as metabolite accumulation [21] muscle glycogen depletion [22,23], and fat oxidation [24]. Currently, data on the metabolomic alterations that occur during exercise are still limited. A previous study of healthy adults subjected to submaximal exercise showed significant increases in a range of purine metabolites and several acyl carnitines [25]. Understanding of such metabolic changes could enable the elucidation of individual’s ability to maintain peak performance and physiological function [20].

When looking at the traditional marathon, physical performance can be affected by gender [26,27,28], age [29], lifestyle, and body mass index (BMI) [30], through differences in physiological (e.g., muscle strength, oxygen carrying capacity) and morphological (e.g., percentage of body fat, muscle mass) characteristics of an individual [31]. However, the amount of exercise optimal for a given individual remains unknown due to absence of definitive data on the molecular mechanisms underlying exercise in relation to health, particularly regarding ultra-endurance distances. Therefore, investigation of the metabolomic effect of exercise on the human metabolome could provide insights into phenotypic responses, permit development of personalized training regimes based on initial metabolic status of an individual [32], and yield vital diagnostic and prognostic biomarkers for use by physicians in the management of cardiovascular and other related diseases [33].

The aim of the current study was to analyze the change in metabolic profile of trained ultramarathon runners in response to an 80.5 km simulated treadmill ultramarathon in a controlled laboratory environment. Plasma samples were analyzed for their metabolomic profiles to determine the metabolic changes due to extreme exercise in order gain some insight into how metabolism is adapted for endurance performance.

## 2. Results

### 2.1. Physiological Response to the Ultramarathon

Nine male participant runners (mean age 34 ± 7 years, $\dot{V}$O_2max_ 61.6 ± 4.3 mL/min/kg) completed the time trial in 9 h:17 min:18 s ± 1 h:18 min:24 s. The fastest time to complete was 7 h:4 min:9 s and the longest time to complete was 10 h:37 min:37 s with an average $\dot{V}$O_2max_ as percentage of maximum (64.5 ± 3.8%).

### 2.2. Variation of Metabolic Profile with Exercise

**Figure 1:**
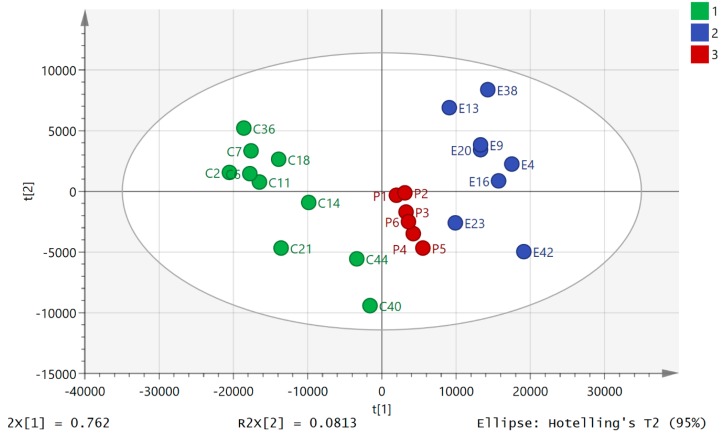
PCA separation of pre-80.5 km samples (C, *n* = 9) and post 80.5 km (E, *n* = 8) samples based on 446 ^13^C_2_ glycine polar metabolites analyzed on a ZICpHILIC column (where *p* = pooled samples, *n* = 6). One post sample in the set is missing due to a technical failure. The data was Pareto scaled and log transformed.

**Figure 2:**
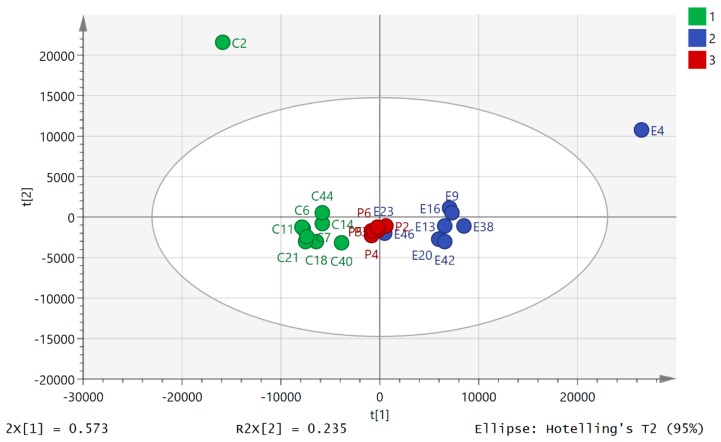
PCA separation of pre-80.5 km samples (C, *n* = 9) and post 80.5 km (E, *n* = 9) samples based on 300 lipophilic metabolites analyzed on an ACE C4 column (where *p* = pooled samples, *n* = 6). The data was Pareto scaled.

Principal component analysis (PCA) of the HILIC data showed clear separation between the pre- and post-80.5 km samples (Figure 1). The data set of 542 polar metabolites was filtered by excluding 96 metabolites which had relative standard deviation (RSD) values >20% within the pooled samples. Figure 1 shows a clear separation of the pre- and post 80.5 km samples according to principial component analysis (PCA) based on 446 metabolites annotated to Metabolomics Standards Initiative (MSI) levels 2/1 [34], where level 2 consisted of mass matching to <3 ppm to a metabolite in the database and level 1 consisted of mass matching to <3 ppm and a retention time corresponding to a standard. The pooled samples (P) clustered in the middle of the plot indicating reasonable technical stability throughout the run (Figure 1, P1–6) but they indicated some drift which may account to some of the variation between individuals. There was a technical problem with one of the post-80.5 km samples which was removed from the plot (total post 80.5 km *n* = 8). The model explained 82.6% of the variation in the data in principal component analysis score plot (PC1 and PC2). From Figure 1 it can be seen that ultramarathon running has a strong impact on the levels of polar metabolites in plasma although there is considerable variation between with individuals with regard to their response. The data for the 500 lipophilic metabolites was filtered by excluding 200 metabolites which had RSD >20% in the pooled samples. The PCA model shown in Figure 2 is based on 300 metabolites annotated to MSI level 2. The model explains 80.7% of the variation in the data in two components. It is not as strong as the model based on polar metabolites and pre-80.5 km samples C2 and E2 are outliers taken from the same individual. Figure S1 shows that there was no separation between two baseline samples one having been taken prior to the day of the run for a model based on the polar metabolites. Figure S2 shows that there was no clear separation between the two sets of baseline samples although again sample B1 is an outlier for the same individual who produced outliers in Figure 2. Figures S3–S80 show comparisons of the absolute response in pre-80.5 km and post-80.5 km for selected metabolites using bar graphs along with comparison of these metabolites in baseline and pre-80.5 km. It is clear that the marked shifts in metabolite levels between the pre-80.5 km and post-80.5 km samples are absent when baseline and pre-80.5 km samples are compared. The only metabolites that show some change between baseline and post-80.5 km are bile acids. We observed previously that bile acids show some diurnal variation [25].

### 2.3. Univariate Comparisons

As can be seen in Table 1 there were a very large number of metabolic changes resulting from the bout of ultramarathon running, with many amino acids decreasing in abundance while there were increases in the levels of many acylcarnitines, fatty acids and oxidized fatty acids. In many cases, the findings are significant for the comparison of the pre- and post-80.5 km samples and a false discovery rate (FDR) test confirmed the significance to all metabolites with *p* < 0.05 [35]. In order to gain a comprehensive overview, analysis was also carried out by RP chromatography, which was useful for getting a clearer picture of the lipophilic compounds in plasma including long-chain acylcarnitines, fatty acids and oxidized fatty acids. The results from the RP analysis of acylcarnitines, fatty acids and oxidized fatty acids are also shown in Table 1. The RP mode was better for these classes of compounds since in HILIC mode they all eluted close to the column void volume. Table S1 shows the metabolites, which were matched against a standard. Table S2 shows a list of the standards that were run in five standard mixtures plus a mixture of free fatty acids derived from two fixed oils, olive oil and cod liver oil.

## 3. Discussion

**Figure 3:**
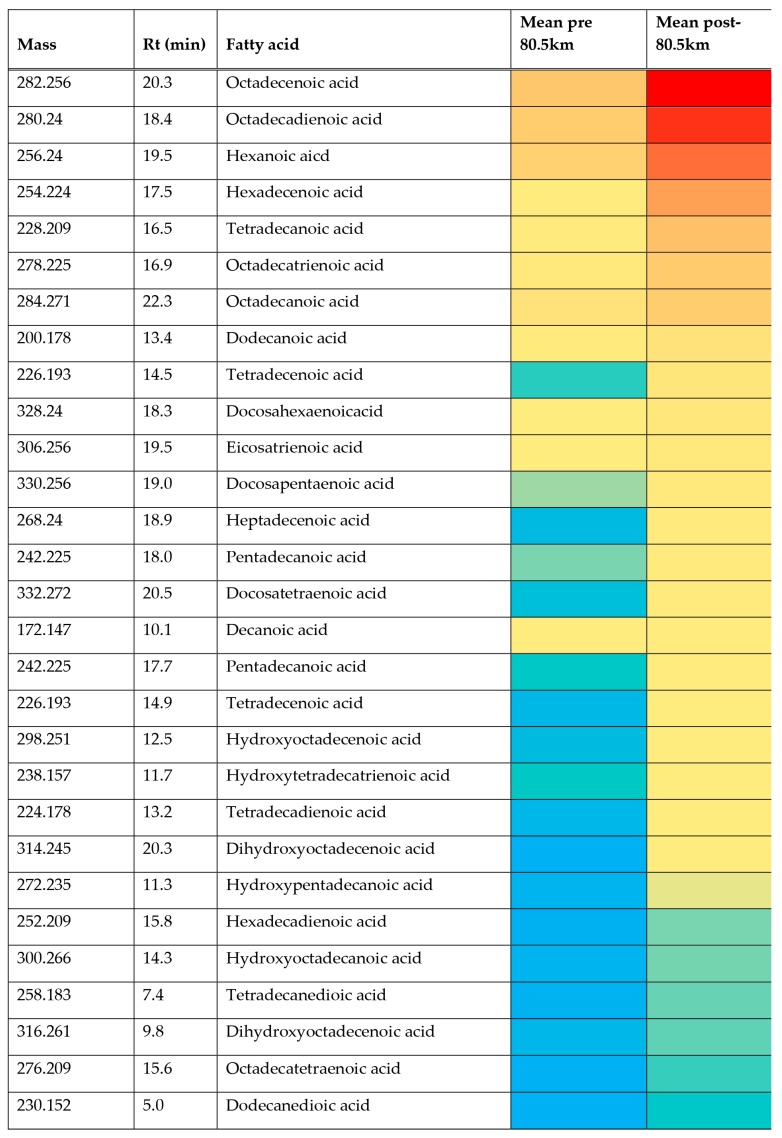
Heat map showing the relative abundance of the 30 most abundant fatty acids in plasma for the pre- and post-80.5 km samples and two post-exercise samples. Red = highest value (3.93 × 10^7^), Yellow = 1 × 10^5^ and blue = 5 × 10^3^. Rt = retention time.

The aim of the current study was to determine an untargeted metabolic response to an 80.5 km treadmill-based ultramarathon. The observed clear separation between baseline samples (pre 80.5 km) and samples taken immediately on completion of the distance (post 80.5 km) with the use of a PCA model demonstrated that there were significant metabolic changes induced by the extreme exercise of ultramarathon running. For some metabolites, the changes were very large and were highly significant when the two cohorts were compared (Table 1). The major changes concern fatty acid metabolism, with a large elevation in acylcarnitine levels in plasma for a wide range of these compounds. The impact of exercise on carnitines has been observed before in a number of studies [36,37,38,39,40]. A possible explanation is that the carnitines reflect mitochondrial fatty acid oxidation as an energy source under the impact of exercise. This is of interest since acylcarnitine accumulation has been identified during prolonged fasting and similar increased demands on stored energy [39] would be expected in an ultra-marathon event. The elevations in fatty acids are supported by previous findings [41] where it was demonstrated that elevated rates of fat oxidation and ability to maintain glycogen concentrations were found in highly trained ultra-endurance athletes. It was shown in the metabolic response to a 24 h ultra-marathon run that there was an increased lipid profile with substrate utilization shift towards fat oxidation [24] suggesting reduction in cardiovascular disease (CVD) risk [42]. More recently it has been hypothesized that acyl carnitines have neuroactive properties that can regulate exertion via interaction with the neurons regulating muscle activity [40]. Less frequently studied are the products of fatty acid oxidation that accumulate in plasma during exercise [43,44]. Many oxidized fatty acids have potent effects on blood vessels promoting either vasodilation or vasoconstriction [45]. As observed (Table 1 and Figure S81) there is a complex mixture of fatty acids all of which are greatly elevated in plasma following exercise. The oxidation products of linoleic acid 9-hydroxylinoleic acid and 13-hydroxylinoleic acid have been proposed as markers of oxidative stress following exercise and several isomers of hydroxylinoleic acids were elevated in the post-80.5 km samples in comparison to baseline (Table 1). This supports the findings of previous studies that have demonstrated ultra-endurance exercise-induced reactive oxygen species (ROS) production [46]. Figure S81 shows extracted ion chromatograms for the pre- and post- levels of oxidized octadecadienoic acid. The range of oxidized fatty acids elevated post-exercise is extensive and the increases very marked (Table 1), therefore the elevation of hydroxyoctadecadienoicacids is not exclusive and there are many other hydroxy acids which are elevated post-exercise plus some dioic acids. Whether or not these acids also have biological activities is unknown, as is the precise reason for their elevation. When the heat map (Figure 3) is considered it is evident that many of the oxidized fatty acids, although elevated (Table 1), are of relatively low abundance. It has been suggested that oxidized acids are a marker of oxidative stress [43,44] but it might be expected that other readily oxidized acids present in plasma, such as eicosapentaenoic acid (EPA), might also be oxidized in the same way, but despite EPA being relatively abundant in the plasma, no peaks for hydroxy EPAs can be seen. Therefore, it is possible that there is some biological mechanism that keeps oxidation products of EPA at low levels since many of these metabolites have potent anti-inflammatory and vasoactive effects [47]. Although oxidative stress was not measured in the current study, previous studies have demonstrated ultra-marathon running induces reactive oxygen species (ROS) production and markers of oxidative damage [48] and this warrants further investigation.

**Figure 4:**
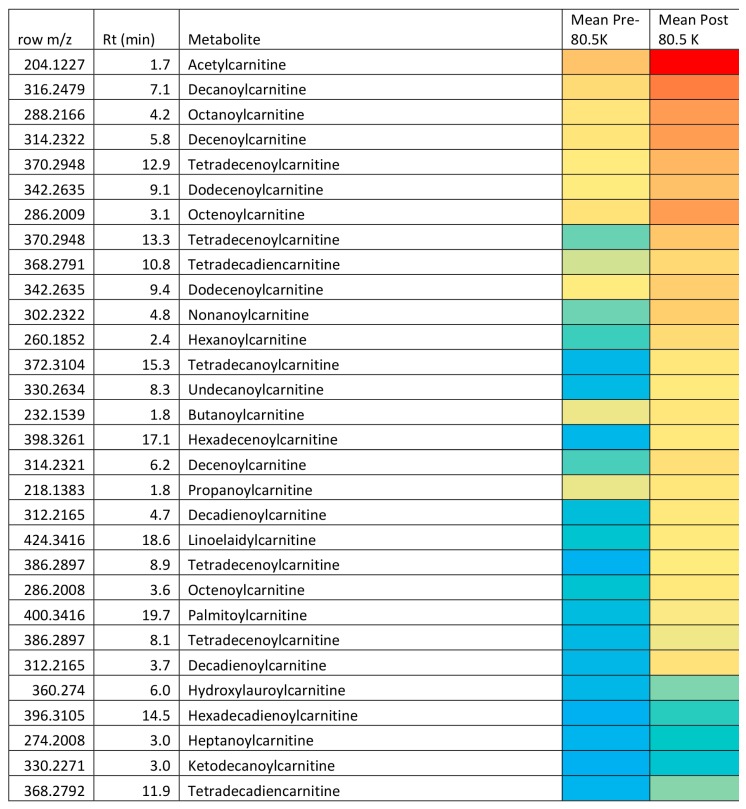
Changes in the 40 most abundant acylcarnitines in plasma following an ultramarathon analyzed by RP method for the pre- and post-80.5 km samples and two post-exercise samples. Red = highest value (2.6 × 10^7^), Yellow = 5 × 10^4^ and blue = 5 × 10^3^.

Given the wide range of unsaturated fatty acids and hydroxylated fatty acids (Table 1) it would seem likely that these compounds arise from peroxisomal metabolism and this might provide a protective mechanism for ensuring that the levels of oxidized long-chain unsaturated acids are kept at low levels. Peroxisomes are known to be responsible for degrading prostaglandins [49]. Unlike mitochondrial beta-oxidation of fatty acids, peroxisomal beta-oxidation of fatty acids does not necessarily go to completion and acids may only be shortened by 3–4 cycles of 2 carbon chain shortening [49] yielding a molecule of acetyl CoA/acetyl carnitine at each cycle. For instance, it might be significant that hexadecadienoic acid, tetradecadienoic acid and dodecadiencoic acid are all elevated, these are not abundant naturally occurring fatty acids, but they are all products of chain shortening of linoleic acid via beta-oxidation [49]. Similarly, hexdecatrienoic acid could arise from chain shortening of linolenic acid via one beta-oxidation step. The reason for the metabolism pausing when a double bond is encountered within the fatty acid chain is that at this point further metabolism requires the commitment of nicotinamide adenine dinucleotide phosphate, (NADPH) in the reduction of the double bond before further chain shortening can occur [49]. Under conditions of aerobic stress there will be generally a high requirement for NADPH in countering oxidative stress; it is required for instance in the recycling of glutathione disulfide back to glutathione. The elevated levels of acylcarnitines are consistent with increased beta-oxidation of fatty acids by peroxisomes since they are the major product exported out of peroxisomes resulting from fatty acid beta-oxidation. It has previously been demonstrated that physical exercise increases peroxisome levels in rat heart [50]. Acetyl carnitine is readily utilized by mitochondria as a source of acetyl CoA, which can be metabolized via the Krebs cycle. The major question with regard to carnitines is; are they waste products or utilizable as substrates for further oxidation? Conversion of acylCoAs to acylcarnitines is necessary in order to preserve free levels of CoA within the mitochondria [51]. The heat map (Figure 3) indicates in terms of absolute abundance that the common dietary fatty acids are much higher in plasma than the unusual acids, which are promoted by exercise observed in the current study. Thus, it seems probable that medium chain length unsaturated fatty acids are minor metabolites due to partial metabolism of long-chain unsaturated fatty acids by peroxisomes providing an additional source of acetylcarnitine for export to mitochondria. The heat map (Figure 4) shows the relative abundance of the 40 most abundant acyl carnitines in plasma. Acetyl carnitine is highly abundant while the carnitines corresponding to the medium-chain fatty acids are of much lower abundance. Although the levels of some acyl carnitines rise in urine post-exercise they do not increase to the same extent as the plasma levels in the current study and no increase in post-exercise urinary acetyl carnitine was observed previously [25]. This suggests that the carnitines may be produced for utilization as energy substrates. Conversion of free fatty acids to acyl CoAs requires the investment of a molecule of adenosine triphosphate (ATP). However, acyl carnitines are an activated form of fatty acid substrate and are convertible into acyl CoAs without the investment of ATP in creating the thioester bond and thus they can be taken up into mitochondria and further metabolized [51,52].

Therefore, the pattern of fatty acids and carnitines observed in the current study points strongly towards a large increase in peroxisomal metabolism. For example, a widely studied substrate of peroxisomal metabolism is phytanic acid, which is present in dairy products [50]. This compound undergoes α-oxidation in the peroxisomes producing pristanic acid, which is then further metabolized by the peroxisomes yielding propanolyl CoA (carnitine) and dimethyl nonanoyl CoA (undecanoyl carnitine) after six cycles of beta oxidation. Both of these carnitines are elevated in the post-80.5 km samples and provide potential substrates for mitochondrial metabolism in the muscles.

The increased activity of the peroxisomes is further underlined by elevated levels of some dioic acids (Table 1), which are also only produced by peroxisomes. The hypothesis that the metabolite patterns are consistent with peroxisomal proliferation is consistent with our earlier observations where it was proposed that exercise increased the proliferation of peroxisome proliferator-activated receptor (PPAR)-γ ligands in plasma [53]. From the current study, these ligands might well be long-chain unsaturated fatty acids, which are substrates for peroxisomal metabolism as discussed above.

The levels of almost all the amino acids in the plasma samples decreased significantly. The fall in the amino acids used in protein biosynthesis might be due to an increase in protein biosynthesis during exercise, which was observed to occur [54,55]. Hydrocortisone is responsible for maintaining a homeostasis under stress conditions, in this study both hydrocortisone and its metabolite urocortisone are increased and this was observed to occur in previous studies [56,57]. The most studied metabolites with regard to the effect of exercise and the determination of fitness are metabolites in the purine pathway such as hypoxanthine and inosine; a marked change in levels of hypoxanthine was observed in the present study. The re-uptake of hypoxanthine into muscle was observed to be more efficient in highly trained individuals [58] and the elevation of hypoxanthine in plasma during exercise is less marked than we observed in urine samples taken post-exercise [25]. However, since the athletes in the current study were trained, it might be expected that their metabolism be geared to conserving purines [59]. Future research is also warranted to determine the long-term adaptations to ultramarathon training. Changes in uridine following exercise have been observed previously and most often increases have been observed, in the current case there was a marked decrease [60]. Changes in tocopherols have also been observed previously in exercise studies and γ-tocopherol has been correlated to $\dot{V}$O_2max_ level [37]. In summary, many amino acids were lowered in plasma post-exercise but the clearest impact of endurance exercise is on fatty acid metabolism but with respect to formation of medium-chain unsaturated and partially oxidized fatty acids and conjugates of fatty acids with carnitines. Many of these metabolites were increased several fold. The most likely explanation for the complex pattern of medium-chain and oxidized fatty acids formed is that the prolonged exercise provoked the proliferation of peroxisomes. The peroxisomes may serve two functions, one of providing a readily utilizable form of energy through formation of acetyl carnitine and other acyl carnitines for export to mitochondria in the muscles; which can utilize these substrates without investment of the adenosine triphosphate (ATP) required to conjugate free fatty acids to acetyl-Coenzyme-A. Secondly the peroxisomes may serve to regulate the levels of oxidized metabolites of long-chain fatty acids since many of these metabolites can provoke biological responses such as vasoconstriction or have pro-inflammatory activity.

To the authors’ knowledge, this is the first study to provide evidence of the metabolic profile in response to prolonged ultramarathon running using an untargeted approach. The findings provide an insight to the effects of ultramarathon running on the metabolic specificities and alterations that may demonstrate cardio-protective effects.

## 4. Materials and Methods

### 4.1. Chemicals and Solvents

High-performance liquid chromatography (HPLC) grade Acetonitrile (ACN) was purchased from Fisher Scientific (Loughborough, UK) and HPLC grade water was produced by a Direct-Q3 UltrapureWater System (Millipore, Watford, UK). AnalaR-grade formic acid (98%) was obtained from BDH-Merck (Poole, UK). Authentic stock standard metabolites (Sigma-Aldrich, Poole, UK) were prepared as previously described [61] and diluted four times with ACN LC-MS analysis of the four mixtures of standards (Table S2). Mixtures of fatty acid standards were derived from the hydrolysis of olive oil and cod liver oil with 1 M ethanolic KOH. These oils have well defined fatty acid compositions [62]. Ammonium acetate was purchased from Sigma-Aldrich (Poole, UK).

### 4.2. Participants

Nine healthy trained male participants (mean ± SD) age 34 ± 7 years, $\dot{V}$O_2max_ 61.6 ± 4.3 mL/min/kg, body mass 70.4 ± 6.6 kg, stature 178.3 ± 3 cm, body mass index 22.1 ± 1.7 kg/m^2^ were voluntarily recruited and provided written informed consent. Ethical approval was obtained from Kingston University Faculty Ethics Committee and was conducted in accordance to the declaration of Helsinki. All participants reported no illness or infection in the two weeks leading up to the trial. Participants had on average 5.3 years’ (range 1–25 years) experience in ultra-endurance exercise.

### 4.3. Experimental Design

The study was a cross-sectional observational time-trial conducted at Kingston University London Human Performance Lab and all testing commenced at 07:00 ± 1:00 h. Food and drink was provided ad libitum during the entire duration and self-selected according to the participants preference to replicate habitual ultra-running conditions, but was not considered in the current analysis. However, further analysis of food and fluid consumption may be warranted in further investigations. Participants were asked to refrain from exercise and the consumption of alcohol 24 h prior to commencement of the exercise trial, however were not required to be fasted to replicate habitual ultra-marathon running conditions. 

Plasma samples collected before (pre-80.5 km), and immediately after completion of the distance (post-80.5 km) were analyzed for their metabolomic profiles using both HILIC and RPLC-MS methods. 

### 4.4. Blood Sampling

Plasma samples were obtained from venous whole blood collected via venepuncture at rest before commencement of the trial (pre-80.5 km) and on completion of the distance (post-80.5 km). An additional 3 h fasted blood sample (baseline) was also collected at rest from all participants two weeks prior to the 80.5 km trial. This was to enable comparison between baseline (B) and pre-80.5 km (C) samples to validate that the changes seen were in response to the exercise trial (Supplementary Materials Figures S1 and S2). Participants rested in a supine position for 10 min before blood sampling at rest, and supine position immediately on completion of the trial. Blood samples were collected by venepuncture from an antecubital vein of the forearm using EDTA vacutainers (Becton, Dickinson and Company, Plymouth, UK). Blood samples were immediately centrifuged for 10 min at 2000 *g* at 4 °C and plasma aliquots stored at −80 °C for subsequent analysis.

### 4.5. Sample Preparation

Exactly 100 µL of plasma was mixed with 400 µL of acetonitrile containing 5 µg/mL of ^13^C_2_ glycine (Sigma-Aldrich, Poole, UK) as an internal standard to ensure retention time stability, and then centrifuged for 10 min before transferring into a vial with an insert. The pooled sample was prepared by pipetting 50 µL from each of the 46 samples and then mixing them together before being diluting 0.2 mL of the pooled sample with 0.8 mL of acetonitrile containing 5 µg/mL ^13^C_2_ glycine internal standard followed by centrifuging. Additionally, the prepared mixtures of authentic standard metabolites [61] containing 5 µg/mL of ^13^C_2_ glycine as internal standard were run. 

### 4.6. LC-MS Conditions

Liquid chromatographic separation was carried out on an Accela HPLC system interfaced to an Exactive Orbitrap mass spectrometer (Thermo Fisher Scientific, Bremen, Germany) using both a ZIC-pHILIC column (150 × 4.6 mm, 5 µm, HiChrom, Reading, UK) and a reversed phase column (ACE C4, 150 × 3.0 mm, 3 µm, HiChrom, Reading, UK). The metabolites were eluted from the ZICpHILIC column with a mobile phase consisting of 20 mM ammonium carbonate in HPLC-grade water (solvent A) and acetonitrile (solvent B), at a flow rate of 0.3 mL/min. The elution gradient was an A:B ratio of 20:80 at 0 min, 80:20 at 30 min, 92:8 at 35 min and finally 20:80 at 45 min. The mobile phase for elution of the ACE C4 column consisted of 1 mM acetic acid in water (A) and 1 mM acetic acid in acetonitrile (B), at a flow rate of 0.4 mL/min. The elution gradient was as follows: A:B ratio 60:40 at 0 min, 0:100 at 30 min, 0:100 at 36 min, 60:40 at 37 min, 60:40 at 41 min. The nitrogen sheath and auxiliary gas flow rates were maintained at 50 and 17 arbitrary units. The electrospray ionization (ESI) interface was operated in both positive and negative modes. The spray voltage was 4.5 kV for positive mode and 4.0 kV for negative mode, while the ion transfer capillary temperature was 275 °C. Full scan data were obtained in the mass-to-charge range of 75 to 1200 amu for both ionization modes. The MS system fully calibrated prior to running according to manufacturer’s guidelines. The resulting data were acquired using the XCalibur 2.1.0 software package (Thermo Fisher Scientific, Bremen, Germany). The samples were run pairwise with two pooled samples being run at the beginning two after ten samples and then two at the end.

### 4.7. Data Extraction and Analysis

The data was extracted by using MZ Match software (http://mzmatch.sourceforge.net/) [63] and the identification of putative metabolites was made via the macro-enabled Excel file, Ideom (http://mzmatch.sourceforge.net/ideom.html). The lists of the metabolites obtained from these searches were then carefully evaluated manually by considering the quality of their peaks and their retention time match with the standard metabolite mixtures run in the same sequence. All reported metabolites were within 3 ppm of their exact masses. Statistical analyses were performed using both univariate with Microsoft Excel and multivariate approaches using SIMCA-P software version 14.1 (Umetrics, Umea, Sweden). All subsequent metabolite responses were compared by paired *t*-test using Microsoft Excel in order to indicate significant differences, where *p* < 0.05. Multivariate data analysis was employed with SIMCA by fitting PCA-X models having refined the metabolite lists by removing metabolites with RSD values >20% in the pooled samples.

## 5. Conclusions

The clearest impact of endurance exercise is on fatty acid metabolism but with respect to formation of medium-chain unsaturated and partially oxidized fatty acids and conjugates of fatty acids with carnitines. The most likely explanation for the complex pattern of medium-chain and oxidized fatty acids formed is that the ultramarathon provoked the proliferation of peroxisomes. The peroxisomes may serve two functions, one of providing a readily utilizable from of energy in the form of acetyl carnitine and other acyl carnitines for export to mitochondria in the muscles, without the investment of the ATP required to conjugate free fatty acids to CoA. Secondly the peroxisomes may serve to regulate the levels of oxidized metabolites of long-chain fatty acids since many of these metabolites can provoke biological responses such as vasoconstriction or have pro-inflammatory activity. This is the first study using an untargeted metabolomics approach to determine the metabolic profile in response to ultramarathon running. The findings provide an insight to the effects of ultramarathon distance running on the metabolic specificities and alterations that may demonstrate cardio-protective effects.

## Figures and Tables

**Table 1:** All the metabolites affected significantly by the ultramarathon time trial (*p* value < 0.05) or fold change >2 or <0.5. * Matches retention time of standard. ^‡^ Data from runs on ACE C4 column otherwise run on the pHILIC column. A separate list of metabolites identified at MSI level 1 is given in Table S1.

Mode	Mass	RT (min)	Metabolite	Ratio [Post-80.5 km/Pre-80.5 km]	*p*-Value
			**Amino acids and their metabolites**		
+	75.032	15.4	* Glycine	0.510	<0.001
+	89.048	14.4	* Alanine	0.603	0.012
+	103.063	13.4	* 3-Amino-isobutanoate	0.392	<0.001
+	105.043	15.7	* Serine	0.512	<0.001
+	111.032	9.5	Pyrrole-2-carboxylate	0.413	<0.001
+	115.063	12.4	* Proline	0.420	<0.001
+	116.047	1.7	Oxopentanoic acid	0.819	<0.001
+	117.054	15.5	Guanidinoacetate	0.627	0.001
+	117.079	12.1	* Valine	0.447	<0.001
+	117.079	10.8	* Betaine	0.505	<0.001
-	118.063	1.8	Hydroxypentanoate	1.393	<0.001
+	119.058	14.4	* Threonine	0.217	<0.001
+	125.015	15.4	* Taurine	0.565	0.001
+	129.043	14.1	5-Oxoproline	0.352	<0.001
+	131.058	14.1	Hydroxyproline	0.361	<0.005
+	131.095	10.3	* Leucine	0.455	<0.001
+	131.095	10.8	* Isoleucine	0.430	<0.001
+	132.079	2.1	Hydroxyhexanoic acid ^‡^	2.237	0.004
+	132.053	15.2	* Asparagine	0.465	<0.001
+	132.090	22.4	* Ornithine	0.545	0.003
+	138.043	8.8	* Urocanate	0.626	0.019
+	146.069	14.8	* Glutamine	0.710	<0.001
+	146.106	23.8	* Lysine	0.369	<0.006
+	147.053	11.2	* Glutamate	0.528	<0.001
+	149.051	11.2	* Methionine	0.609	<0.003
-	154.038	11.7	Imidazol-5-yl-pyruvate	0.469	<0.001
-	159.068	8.1	Indole-3-acetaldehyde	0.432	0.001
+	161.069	9.9	O-Acetylhomoserine	0.524	<0.001
+	174.112	25.4	* Arginine	0.387	<0.003
+	175.096	15.6	* Citrulline	0.673	0.047
+	181.074	12.8	* Tyrosine	0.761	0.016
-	182.058	9.4	Hydroxyphenyllactate	0.541	0.002
+	188.116	16.2	N6-Acetyl-l-lysine	0.233	0.054
+	189.043	6.4	Kynurenate	2.322	0.001
+	204.090	11.1	* l-Tryptophan	0.539	<0.001
+	208.085	10.2	Formylhydroxykynurenamine	0.668	0.004
-	219.053	4.9	Hydroxyindolepyruvate	5.131	0.010
			**Acylcarnitines**		
+	204.123	10.3	* Acetylcarnitine	3.353	<0.001
	218.138	9.1	Propanoylcarnitine	1.420	0.042
+	232.154	7.9	Butanoylcarnitine	1.775	0.010
+	258.170	2.1	Hexenoylcarnitine ^‡^	6.350	0.002
+	260.185	2.4	* Hexanoylcarnitine isomer ^‡^	9.640	0.011
	260.185	2.9	Hexanoylcarnitine isomer ^‡^	13.091	0.045
+	274.201	3.0	Heptanoylcarnitine ^‡^	5.685	0.013
+	286.201	3.6	Octenoylcarnitine ^‡^	6.009	0.003
+	286.201	3.1	Octenoylcarnitine ^‡^	5.184	0.001
+	288.217	4.2	* Octanoylcarnitine ^‡^	7.119	0.004
	302.232	4.8	Nonanoykcarnitine ^‡^	14.587	0.001
+	312.217	4.7	Decadienoylcarnitine ^‡^	7.016	0.001
+	312.217	3.7	Decadienoylcarnitine ^‡^	16.727	0.102
+	314.232	6.2	Decenoylcarnitine ^‡^	7.186	0.039
+	314.232	5.8	Decenoylcarnitine ^‡^	6.285	0.004
+	316.248	7.1	* Decanoylcarnitine ^‡^	5.017	0.005
	330.227	4.4	Keto-decanoylcarnitine ^‡^	13.121	0.000
+	330.227	3.0	Keto-decanoylcarnitine ^‡^	7.719	0.003
	330.263	8.3	Dimethylnonanoylcarnitine ^‡^	11.088	0.002
+	342.264	9.4	Dodecenoylcarnitine ^‡^	6.439	0.089
+	342.264	9.1	* Dodecenoylcarnitine ^‡^	8.849	0.004
+	360.274	6.0	Hydroxydodecanoylcarnitine ^‡^	4.825	0.003
+	368.279	10.8	Tetradecadiencarnitine isomer ^‡^	5.659	0.022
+	368.279	9.5	Tetradecadiencarnitine isomer ^‡^	24.743	0.012
+	368.279	9.9	Tetradecadiencarnitine isomer ^‡^	19.098	0.055
+	368.279	11.9	Tetradecadiencarnitine isomer ^‡^	9.195	0.031
+	370.295	13.3	Tetradecenoylcarnitine isomer ^‡^	16.422	0.070
+	370.295	12.9	Tetradecenoylcarnitine isomer ^‡^	9.253	0.004
+	372.310	15.3	* Tetradecanoylcarnitine ^‡^	18.265	0.007
+	384.274	6.6	Hydroxytetradecadiencarnitine ^‡^	11.908	0.001
+	386.290	8.1	Hydroxytetradecenoylcarnitine ^‡^	6.193	0.007
+	386.290	8.9	Hydroxytetradecenoylcarnitine ^‡^	27.813	0.003
+	388.305	9.4	Hydroxymyristoylcarnitine ^‡^	4.245	0.006
+	396.310	15.2	Hexadecadienoylcarnitine ^‡^	90.958	0.149
+	396.311	14.5	Hexadecadienoylcarnitine ^‡^	17.816	0.016
+	398.326	17.1	Hexadecenoylcarnitine ^‡^	14.097	0.011
+	400.342	19.7	Palmitoylcarnitine ^‡^	4.618	0.089
+	412.305	9.3	Hydroxyhexadecadienoylcarnitine ^‡^	6.590	0.003
+	414.321	11.1	Hydroxyhexadecenoylcarnitine ^‡^	35.292	0.003
+	424.342	18.6	Octadecadienoylcarnitine ^‡^	3.955	0.048
+	424.342	19.3	Octadecadienoylcarnitine ^‡^	6.043	0.121
+	430.316	8.1	Hexadecanedioicacidmonocarnitineester ^‡^	114,475.436	0.015
			**Fatty acids**		
-	172.147	10.0	Decanoic acid ^‡^	1.909	0.034
-	196.146	10.1	Dodecadienoic acid ^‡^	5.989	0.001
-	200.178	13.4	Dodecanoic acid	4.342	0.009
-	202.120	3.2	Decanedioic acid ^‡^	6.045	0.004
-	210.126	9.3	Hydroxydodecatrienoic acid ^‡^	4.709	0.001
-	212.178	13.3	Tridecenoic acid ^‡^	13.224	0.006
-	224.178	13.2	Tetradecadienoic acid ^‡^	10.003	0.013
-	226.193	14.5	Tetradecenoic acid isomer ^‡^	25.065	0.004
-	226.193	14.9	Tetradecenoic acid isomer ^‡^	14.409	0.020
-	230.152	5.0	Dodecanedioic acid ^‡^	9.432	0.014
-	240.173	8.2	Hydroxytetradecadienoic acid ^‡^	11.109	0.002
-	240.209	16.5	Pentadecenoic acid ^‡^	3.192	0.007
-	242.188	11.4	Hydroxytetradecadienoic acid ^‡^	4.066	0.001
-	244.204	8.2	Hydroxytetradecanoic acid isomer ^‡^	11.109	0.002
-	244.204	9.3	Hydroxytetradecanoic acid isomer ^‡^	3.581	0.000
-	252.209	15.8	* Hexadecadienoicacid isomer ^‡^	10.174	0.059
-	252.209	16.3	* Hexadecadienoicacid isomer ^‡^	13.108	0.041
-	254.224	17.5	* Palmitoleic acid ^‡^	38.719	0.006
-	258.183	7.4	Tetradecanedioic acid ^‡^	7.206	0.006
-	266.188	13.6	Hydroxyhexadecatrienoic acid ^‡^	8.977	0.014
-	268.204	11.3	Hydroxyhexadecadienoic acid	3.355	0.007
-	268.240	18.9	Heptadecenoic acid ^‡^	29.923	0.004
-	270.220	12.1	Hydroxyhexadecenoic acid isomer ^‡^	3.553	0.004
-	270.220	17.5	Hydroxyhexadecenoic acid ^‡^	8.969	0.001
-	272.235	11.3	Hydroxyhexadecanoic acid ^‡^	8.969	0.001
-	276.209	15.6	* Octadecatetraenoic acid ^‡^	10.190	0.067
-	278.225	16.9	* Linolenic acid ^‡^	8.511	0.003
-	280.240	18.4	* Linoleic acid ^‡^	5.769	0.008
-	282.256	20.3	* Oleic acid ^‡^	6.231	0.000
-	284.199	9.1	Dihydroxyhexadecadienoic acid ^‡^	2.897	0.001
-	286.214	10.5	Dihydroxyhexdecenoic acid ^‡^	16.426	0.001
-	296.235	14.0	Hydroxyoctadecadienoic acid ^‡^	3.145	0.024
-	300.266	14.3	Hydroxyoctdecanoic acid ^‡^	6.618	0.015
-	316.261	9.8	Dihydroxyoctadecanoic acid ^‡^	4.040	0.002
-	327.241	7.0	Nitrooctadecenoic acid ^‡^	10.453	<0.001
-	328.240	18.3	* Docosahexaenoicacid ^‡^	4.266	0.022
-	330.256	19.0	* Docosapentaenoic acid ^‡^	9.179	0.003
-	332.272	20.5	Docosatetraenoic acid ^‡^	14.588	0.002
			**Steroids**		
-	362.209	4.5	Hydrocortisone	1.787	0.014
-	364.225	5.0	Urocortisone	3.243	0.003
-	376.298	3.9	Hydroxycholanate	0.315	0.004
-	392.293	4.3	Deoxycholanoic acid	0.361	0.026
-	449.314	4.3	Chenodeoxyglycocholate	0.162	<0.001
-	465.309	4.9	* Glycocholate	0.174	0.003
-	515.291	4.5	Taurocholate	0.275	0.039
-	568.324	7.3	Chenodeoxycholic acid glucuronide	0.311	<0.001
-	612.387	4.5	Cholestane—tetrol-glucuronide	0.443	0.001
			**Miscellaneous**		
+	136.039	9.8	* Hypoxanthine	1.917	0.003
-	244.069	9.5	* Uridine	0.420	<0.001
-	244.070	11.7	Pseudouridine	0.416	<0.001
+	136.064	23.7	* 1-Methylnicotinamide	0.226	0.090
-	164.069	11.8	Rhamnose	0.348	<0.001
+	179.079	10.8	Galactosamine	0.181	<0.001
-	180.064	14.1	Hexose	0.447	<0.001
+	214.132	9.4	Dethiobiotin	1.517	0.002
-	416.366	3.4	gamma-Tocopherol	0.529	<0.001
-	430.381	3.4	Alpha-Tocopherol	0.509	<0.001

## Supplementary Materials

The following are available online at http://www.mdpi.com/2218-1989/8/1/14/s1. Figures S1 and S2: Showing PCA Plots for baseline and pre-80.5 km samples. Figures S3–S80: Bar graphs for selected metabolites comparing of pre- and post-80.5 km samples and baseline and pre-80.5 km samples. Figure S81: Extracted ion traces for hydroxyoctadecadienoic acids in a pre-80.5 km sample and a post-80.5 km sample run on an ACE C4 column. Table S1: showing metabolites annotated to MSI level 1. Table S2: A list of 231 metabolite standards used to characterize the columns.
