# Supplementary material for: Untargeted Metabolomics Profiling of an 80.5 km Simulated Treadmill Ultramarathon
Source: Metabolites. 2018 Feb 13;8(1):14. doi: 10.3390/metabo8010014 (PMC5876003; doi:10.3390/metabo8010014)

Figure S1 Comparison of two sets of baseline samples based on 446 polar metabolites showing no clear separation between samples taken >2 weeks before the trial (B) and immediately before the trial (C).

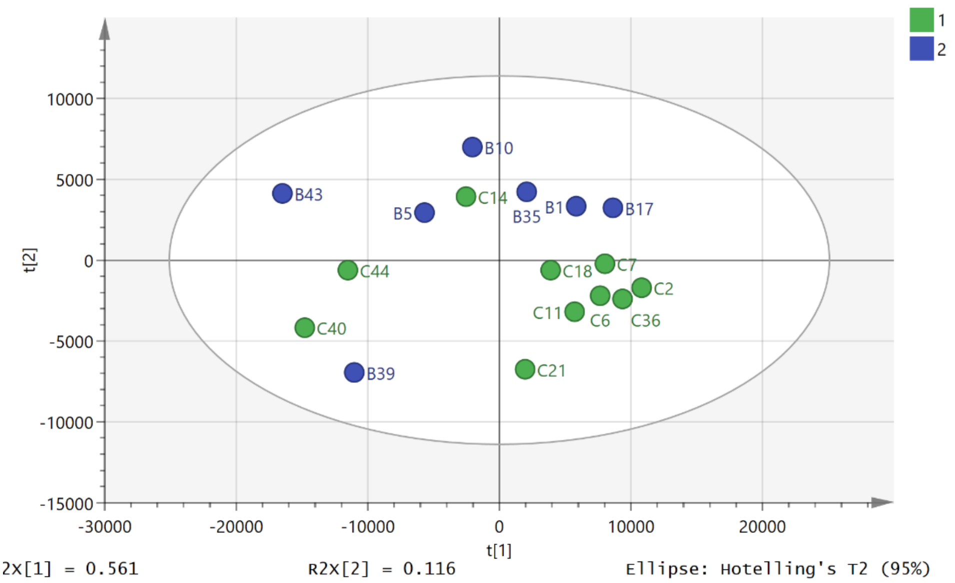

Figure S2 Comparison of baseline samples based on 300 lipophilic metabolites showing no clear separation between samples taken >2 weeks before the trial (B) and immediately before the trial (C).

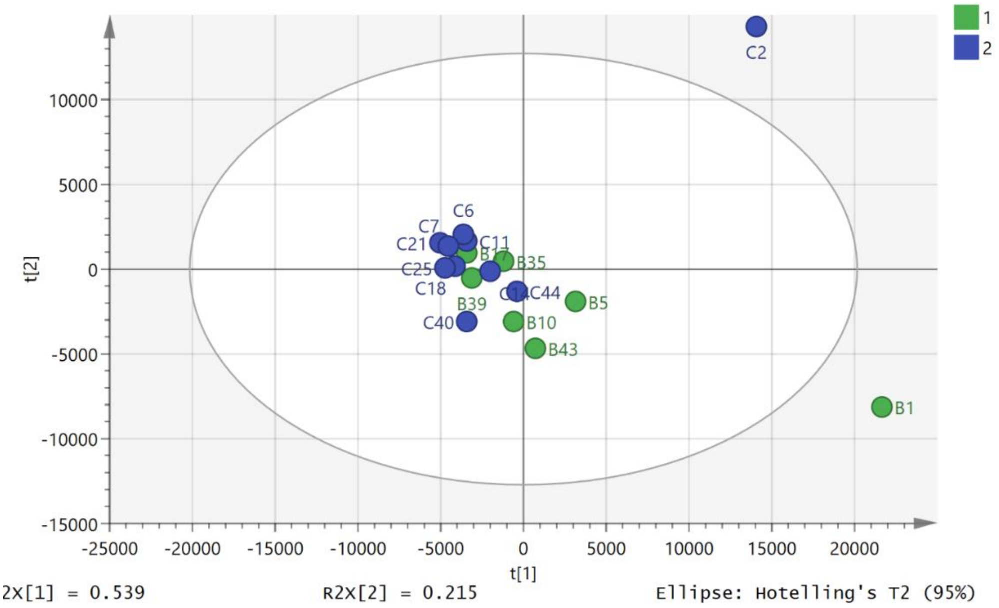

Table S1 Significant metabolites run with matching standard.† Run on ACE C4 columnin comparison with pooled post-80K sample 12 months after original run.

Mass	Rt of std min	Rt in plasma min	
89.0477	14.4	14.4	Alanine
103.063	13.6	13.4	3-Amino-isobutanoate
105.043	15.6	15.7	Serine
115.063	12.3	12.4	Proline
117.079	12.1	12.1	Valine
117.079	10.8	10.8	Betaine
119.058	14.3	14.4	Threonine
125.015	15.3	15.4	Taurine
129.043	14.5	14.1	5-Oxoproline
131.058	14.9	14.1	Hydroxyproline isomer
131.095	10.2	10.3	Leucine
131.095	10.7	10.8	Isoleucine
132.053	15.1	15.2	Asparagine
132.097	22.6	22.6	Ornithine
138.043	8.7	8.8	Urocanate
146.069	14.8	14.8	Glutamine
146.106	24.1	23.8	Lysine
147.053	11.1	11.2	Glutamate
149.051	11.2	11.2	Methionine
165.079	9.6	9.6	Phenylalanine
174.112	25.6	25.4	Arginine
175.096	15.6	15.6	Citrulline
181.074	12.7	12.8	Tyrosine
188.116	14.5	16.2	N6-Acetyl-L-lysine isomer
189.043	7.4	6.4	Kynurenate
204.090	11.1	11.1	Tryptophan
204.1227	10.3	10.3	Acetylcarnitine
136.039	9.8	9.8	Hypoxanthine

244.069	9.5	9.5	Uridine
252.209	16.0	16.0	†Hexadecadienoic acid isomer
252.209	16.8	16.8	†Hexadecadienoic acid isomer
254.225	17.7	17.7	†Palmitoleic acid
260.185	2.8	2.7	†Hexanoylcarnitine
276.210	15.8	15.8	†Octatetraenoic acid
278.225	17.1	17.1	†Linolenic acid
280.239	18.6	18.6	†Linoleic acid
282.256	20.3	20.3	†Oleic acid
288.216	4.3	4.2	†Octanoylcarnitine
316.248	7.0	6.9	†Decanoylcarnitine
328.241	18.4	18.4	†Docosahexenoic acid
330.257	19.2	19.2	†Docasopentaenoic acid
344.278	10.1	10.4	†Dodecanoylcarnitine
372.311	14.1	14.3	†Tetradecanoylcarnitine
465.309	4.5	4.9	Glycocholate
515.291		4.5	Taurocholate

Table S2 List of 231 standards run on the ZICpHILIC and ACE C4 columns.

Compound name	Elemental Composition
Malate	C4H6O5
Phenylethylamine	C8H11N
2-(4-Hydroxyphenyl) ethanol	C8H10O2
2,6-Quinolinediol	C9H7NO2
2-deoxyribose-5-phosphate	C5H11O7P
2-Hydroxybutanoic acid	C4H8O3
2-Indolecarboxylicacid	C9H7NO2
2-Oxoglutarate	C5H6O5
2-phosphoglycerate	C3H7O7P
3-(2-Hydroxyphenyl)propanoic acid	C9H10O3
3-indole acetate	C10H9NO2
3-Phenylpropionylglycine	C11H13NO3
3-Phosphoglycerate	C3H7O7P

4-Coumarate	C9H8O3
4-Hydroxy-L-glutamate	C5H7NO5
4-Hydroxyphenylacetate	C8H8O3
5-Aminolevulinate	C5H9NO3
5-Hydroxyindoleacetate	C10H9NO3
5-methylcytidine	C10H15N3O5
5'-Methylthioadenosine	C11H15N5O3S
5-Oxoproline	C5H7NO3
5-phospho-D-ribose-1-diphosphate	C5H13O14P3
6-Phospho-D-gluconate	C6H13O10P
7-Methylguanosine(+)	C11H15N5O5
Acetoacetate	C4H6O3
Adenine	C5H5N5
Adenosine	C10H13N5O4
ADP	C10H15N5O10P2
Allantoin	C4H6N4O3
AMP	C10H14N5O7P
Amphetamine	C9H13N
Ascorbate	C6H8O6
ATP	C10H16N5O13P3
beta-Alanine	C3H7NO2
beta-D-Fructose-1,6-bisphosphate	C6H14O12P2
Betaine	C5H11NO2
Bilirubin	C33H36N4O6
Biopterin	C9H11N5O3
Biotin	C10H16N2O3S
Caffeate	C9H8O4
Cytidine diphosphate	C9H15N3O11P2
Cholate	C24H40O5
Choline	C5H13NO
Cis-4-Hydroxy-D-Proline	C5H9NO3
Cis-Aconitate	C6H6O6
Citraconate	C5H6O4
Citramalate	C5H8O5
Citrate	C6H8O7
CMP	C9H14N3O8P
CoA	C21H36N7O16P3S
creatine	C4H9N3O2
Creatinine	C4H7N3O
CTP	C9H16N3O14P3
Cystathionine	C7H14N2O4S
Cysteine	C3H7NO2S
Cytidine	C9H13N3O5
Cytosine	C4H5N3O

D(+)2-Phosphoglyceric acid	C3H7O7P
D-2-Hydroxyglutaric acid	C5H8O5
D-3-hydroxy-butyrate	C4H8O3
dAMP	C10H14N5O6P
Deoxyribose	C5H10O4
D-Galacturonate	C6H10O7
D-Gluconic acid	C6H12O7
D-Glucosamine	C6H13NO5
D-Glucosamine 6-Phosphate	C6H14NO8P
D-Glucuronate	C6H10O7
D-Glucuronolactone	C6H8O6
Dihydrobiopterin	C9H13N5O3
Dihydroxyacetone phosphate	C3H7O6P
Dimethyl-L-lysine	C8H18N2O2
D-Isoascorbic acid	C6H8O6
DL-3-aminobutyrate	C4H9NO2
DL-Glyceraldehyde 3-phosphate	C3H7O6P
Dopamine	C8H11NO2
D-Ribose 5-phosphate	C5H11O8P
D-Ribulose 5-phosphate	C5H11O8P
D-Sorbitol	C6H14O6
D-Xylulose 5-phosphate	C5H11O8P
Ectoine	C6H10N2O2
Ethanolamine phosphate	C2H8NO4P
FAD	C27H33N9O15P2
Folate	C19H19N7O6
Fructose	C6H12O6
Fructose 6 phosphate	C6H13O9P
Fucose	C6H12O5
Fumarate	C4H4O4
Galactarate	C6H10O8
galactose	C6H12O6
galactose 1 phosphate	C6H13O9P
Gallate	C7H6O5
GDP	C10H15N5O11P2
Glucose	C6H12O6
Glucose 1 phosphate	C6H13O9P
Glucose 6 phosphate	C6H13O9P
Glutathione	C10H17N3O6S
Glycine	C2H5NO2
Glycocholic acid	C26H43NO6
GMP	C10H14N5O8P
Guanine	C5H5N5O
Guanosine	C10H13N5O5

Hippuric acid	C9H9NO3
Histamine	C5H9N3
Homo-arginine	C7H16N4O2
Homogentisate	C8H8O4
Hypoxanthine	C5H4N4O
Imidazole-4-acetate	C5H6N2O2
IMP	C10H13N4O8P
Indole	C8H7N
Indole-3-lactic acid	C11H11NO3
Inosine	C10H12N4O5
Isocitrate	C6H8O7
Isonicotinic acid	C6H5NO2
Itaconate	C5H6O4
Ketobutyric acid	C4H6O3
Kynurenic acid	C10H7NO3
Lactic acid	C3H6O3
Lactoylglutathione	C13H21N3O8S
L-Adrenaline	C9H13NO3
L-Alanine	C3H7NO2
L-Arabinose	C5H10O5
L-Arginine	C6H14N4O2
L-Asparagine	C4H8N2O3
L-Aspartate	C4H7NO4
L-Carnosine	C9H14N4O3
L-Citrulline	C6H13N3O3
L-Cystine	C6H12N2O4S2
L-Glutamate	C5H9NO4
L-Glutamine	C5H10N2O3
L-Glutathione oxidized	C20H32N6O12S2
L-Histidine	C6H9N3O2
L-Homocysteine	C4H9NO2S
L-Homoserine	C4H9NO3
L-Homoserine Lactone	C4H7NO2
Lipoate	C8H14O2S2
L-isoleucine	C6H13NO2
Lithocholic acid	C24H40O3
L-Kynurenine	C10H12N2O3
L-Leucine	C6H13NO2
L-Lysine	C6H14N2O2
L-Metanephrine	C10H15NO3
L-Methionine	C5H11NO2S
L-Noradrenaline	C8H11NO3
L-Ornithine	C5H12N2O2
L-Phenylalanine	C9H11NO2

L-Proline	C5H9NO2
L-Saccharopine	C11H20N2O6
L-Serine	C3H7NO3
L-Threonic acid	C4H8O5
L-Threonine	C4H9NO3
L-Tryptophan	C11H12N2O2
L-Tyrosine	C9H11NO3
L-Valine	C5H11NO2
Maleic acid	C4H4O4
Malonate	C3H4O4
Maltose	C12H22O11
Mannitol	C6H14O6
mannose	C6H12O6
Melatonin	C13H16N2O2
Mesaconate	C5H6O4
Methyl-L-lysine	C7H16N2O2
Methylmalonate	C4H6O4
N(pi)-Methyl-L-histidine	C7H11N3O2
N6-Acetyl-L-Lysine	C8H16N2O3
N6-Trimethyl-L-lysine	C9H20N2O2
N-Acetyl-D-Glucosamine	C8H15NO6
N-Acetyl-D-glucosamine 6-phosphate	C8H16NO9P
N-Acetyl-D-mannosamine	C8H15NO6
N-Acetylglutamine	C7H12N2O4
N-Acetyl-L-aspartate	C6H9NO5
N-Acetyl-L-glutamate	C7H11NO5
NAD+	C21H27N7O14P2
NADP(+)	C21H28N7O17P3
NADPH	C21H30N7O17P3
N-Formyl-L-methionine	C6H11NO3S
Nicotinamide	C6H6N2O
Nicotinate	C6H5NO2
O-Acetylcarnitine	C9H17NO4
O-Acetylcarnitine(+)	C9H17NO4
O-Acetyl-L-serine	C5H9NO4
Octopamine	C8H11NO2
Oxalate	C2H2O4
Oxypurinol	C5H4N4O2
Pantothenate	C9H17NO5
Paraxanthine	C7H8N4O2
Phenylephrine	C9H13NO2
Phosphocreatine	C4H10N3O5P
Phosphoenolpyruvate	C3H5O6P
Phthalate	C8H6O4

Picolinic acid	C6H5NO2
Putrescine	C4H12N2
Pyridoxal	C8H9NO3
Pyridoxamine	C8H12N2O2
Pyruvate	C3H4O3
Rhamnose	C6H12O5
Riboflavin	C17H20N4O6
ribose	C5H10O5
Ribose 1,5-diphosphate	C5H12O11P2
Ribothymidine	C10H14N2O6
S-Adenosyl-L-homocysteine	C14H20N6O5S
S-Adenosyl-L-methionine(easy degradation)	C15H22N6O5S
Sarcosine	C3H7NO2
Serotonin	C10H12N2O
sn-Glycerol 3-phosphate	C3H9O6P
Spermidine	C7H19N3
Spermine	C10H26N4
Succinate	C4H6O4
Sucrose	C12H22O11
Taurine	C2H7NO3S
Taurocholate	C26H45NO7S
Thiamine	C12H16N4OS
Thymidine	C10H14N2O5
Tryptophanol	C10H11NO
UDP	C9H14N2O12P2
UDP-N-acetyl-D-glucosamine	C17H27N3O17P2
UMP	C9H13N2O9P
Uracil	C4H4N2O2
Urate	C5H4N4O3
Uridine	C9H12N2O6
Uridine 5'-diphosphoglucose	C15H24N2O17P2
Urocanate	C6H6N2O2
UTP	C9H15N2O15P3
Xanthine	C5H4N4O2
Xanthosine	C10H12N4O6
Xylitol	C5H12O5
Xylose	C5H10O5
Hexanoylcarnitine	C13H26NO4
Octanoyl carnitine	C15H30NO4
Decanoyl carnitine	C17H34NO4
Dodecanoyl carnitine	C19H38NO4
Tetradecanoyl carnitine	C21H42NO4

Figures A3-A80 show graphical comparisons between the absolute response for selected metabolites for pre-80K and post 80K samples and baseline and pre-80K samples.

Figure S3 Dodecadienoic acid in pre-80K and post-80K samples

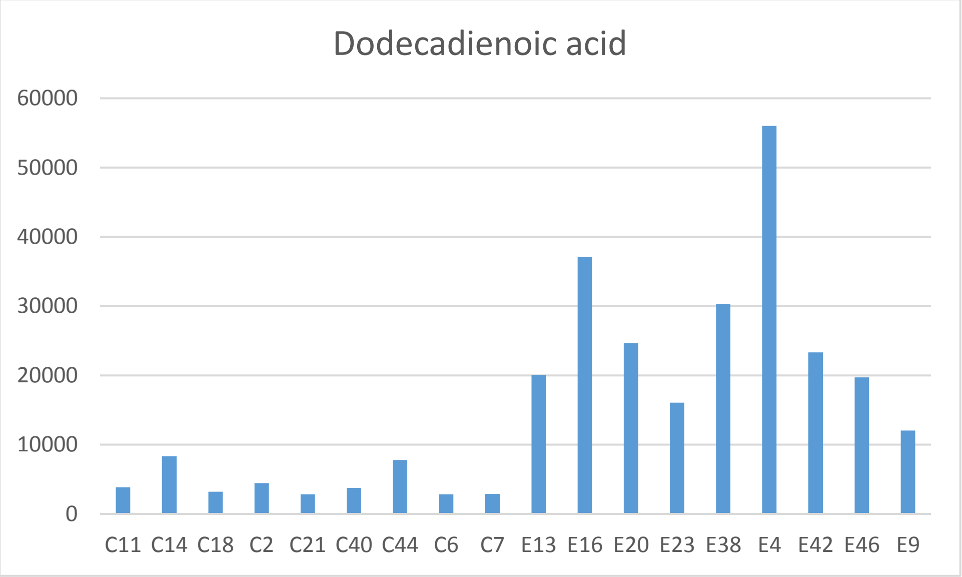

Figure S4 Dodecadienoic acid in baseline and pre-80K samples

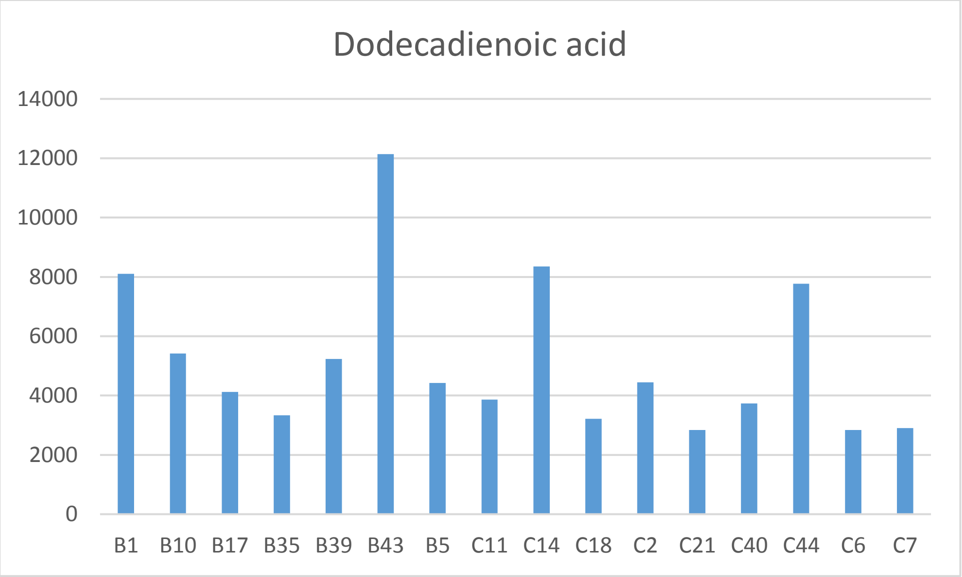

Figure S5 Tridecenoic acid in pre-80K and post-80K samples

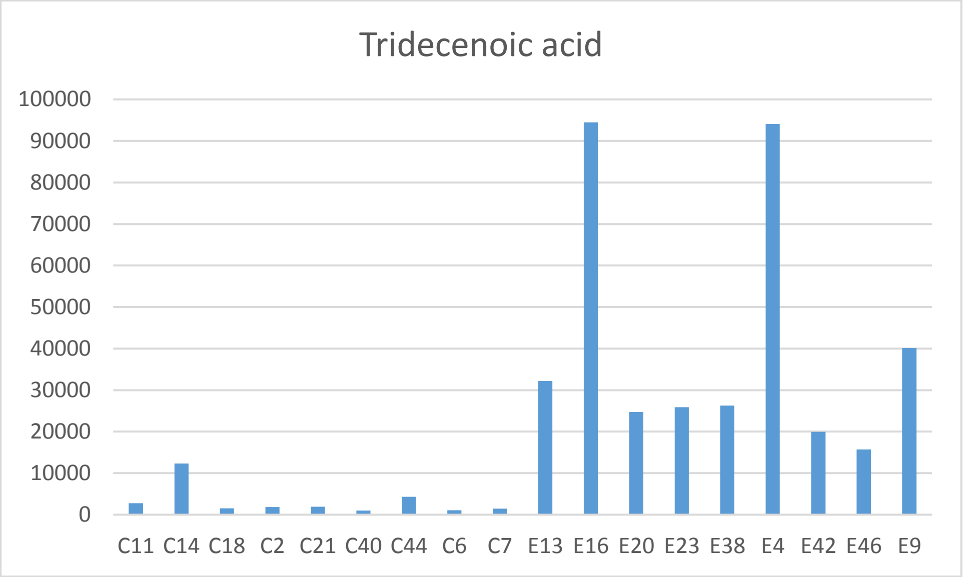

Figure S6 Tridecenoic acid in baseline and pre-80K samples

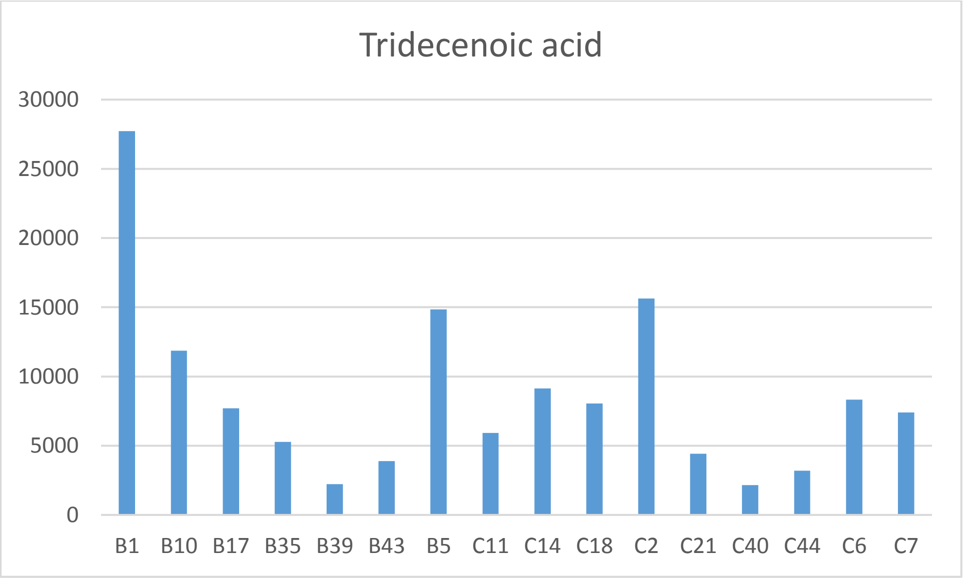

Figure S7 Tetradecadienoic acid in pre-80K and post-80K samples

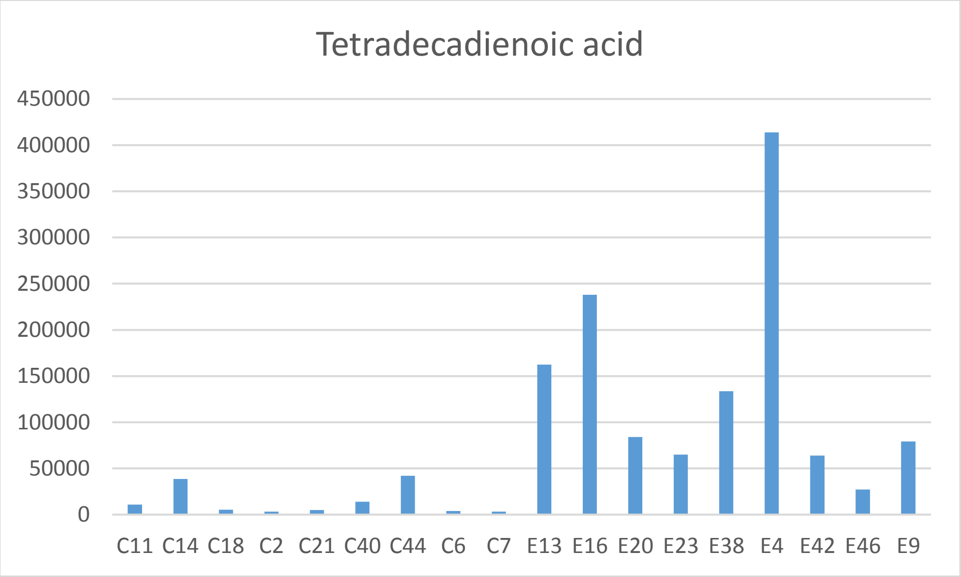

Figure S8 Tetradecadienoic acid in baseline and pre-80K samples

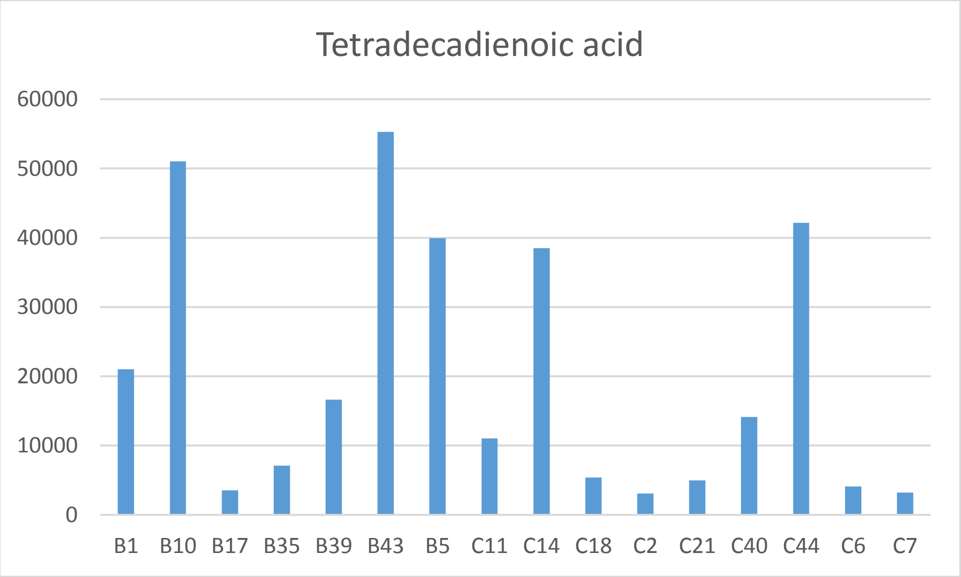

Figure S9 Tetradecenoic acid in pre-80K and post-80K samples

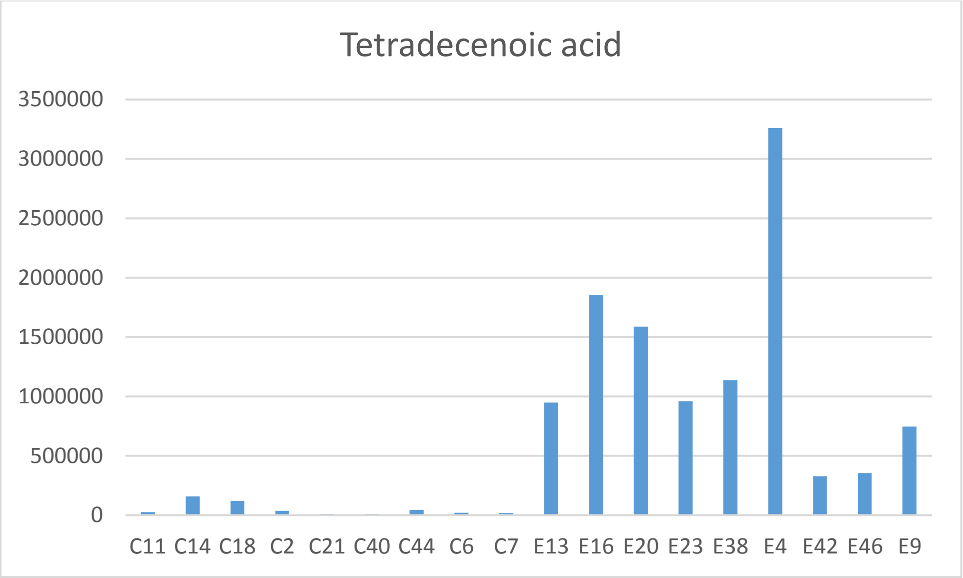

Figure S10 Tetradecenoic acid in baseline and pre-80K samples

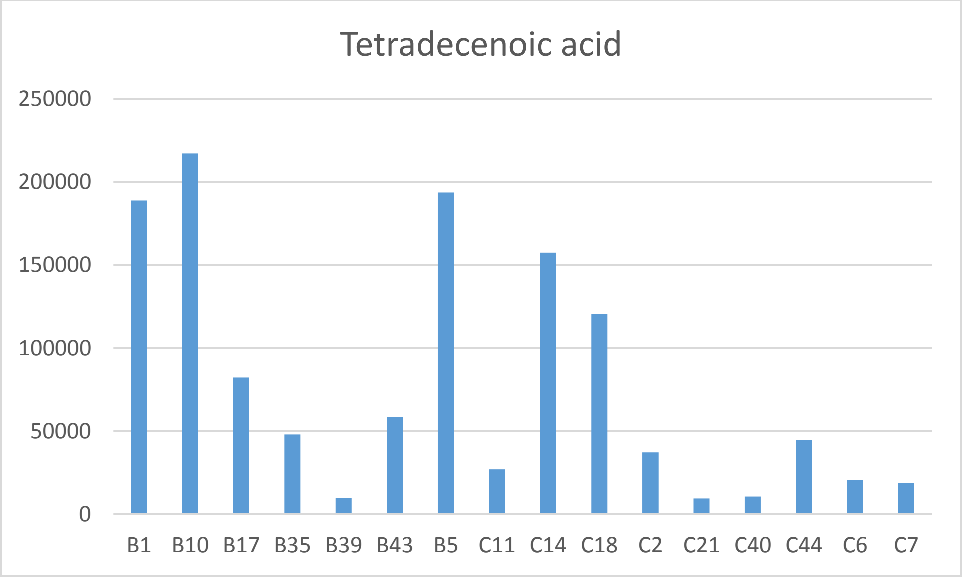

Figure S11 Hexadecadienoic acid in pre-80K and post-80K samples

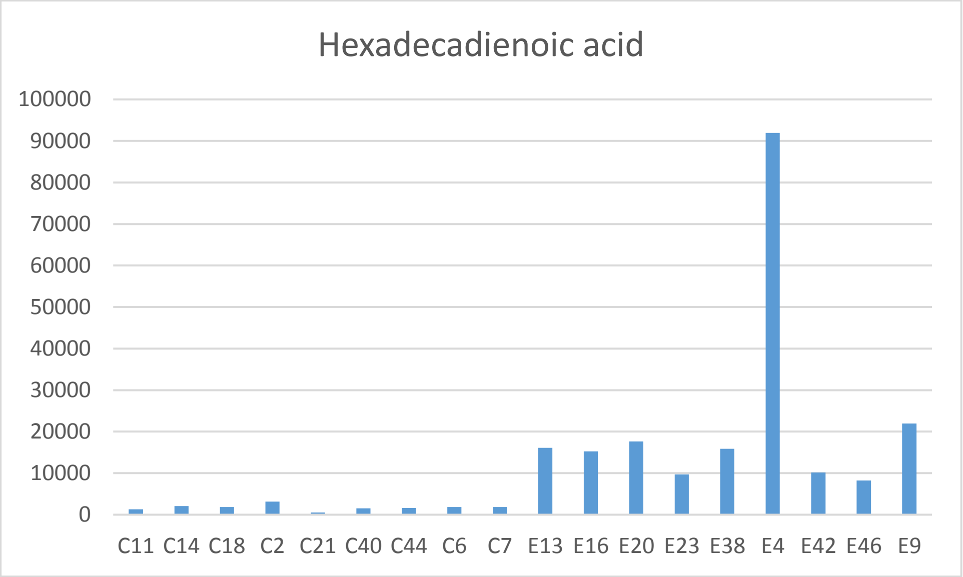

Figure S12 Hexadecadienoic acid in baseline and pre-80K samples

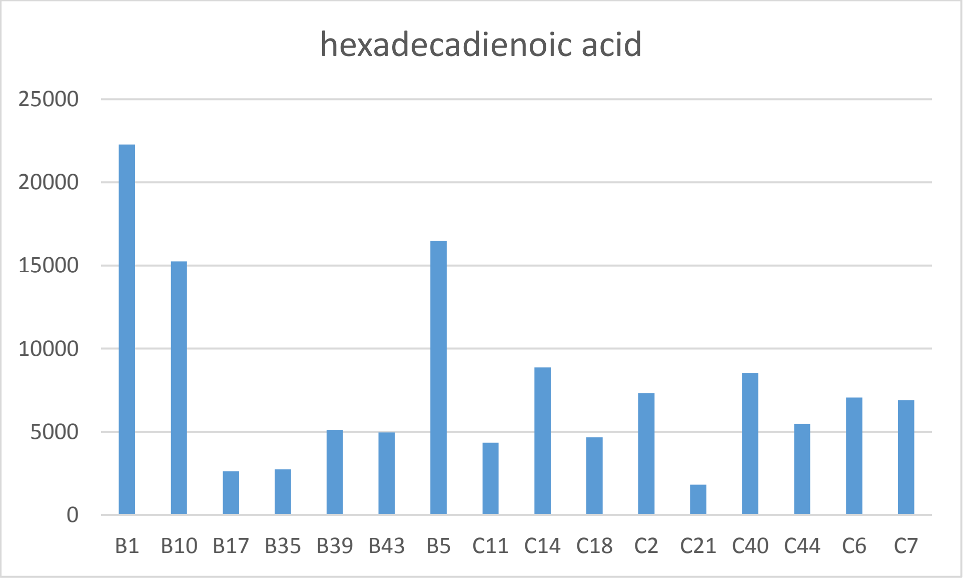

Figure S13 Hexadecenoic acid in pre-80K and post-80K samples

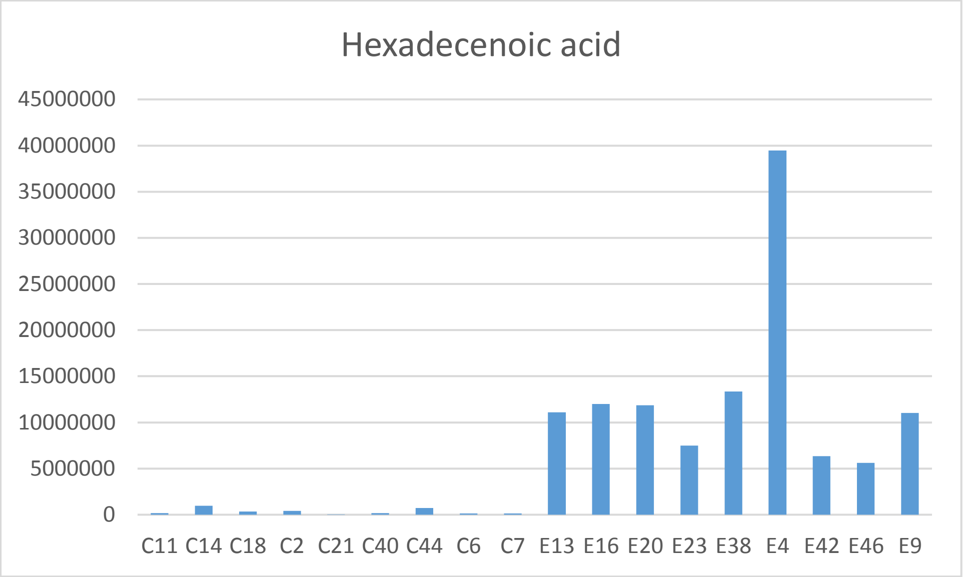

Figure S14 Hexadecenoic acid in baseline and pre-80K samples

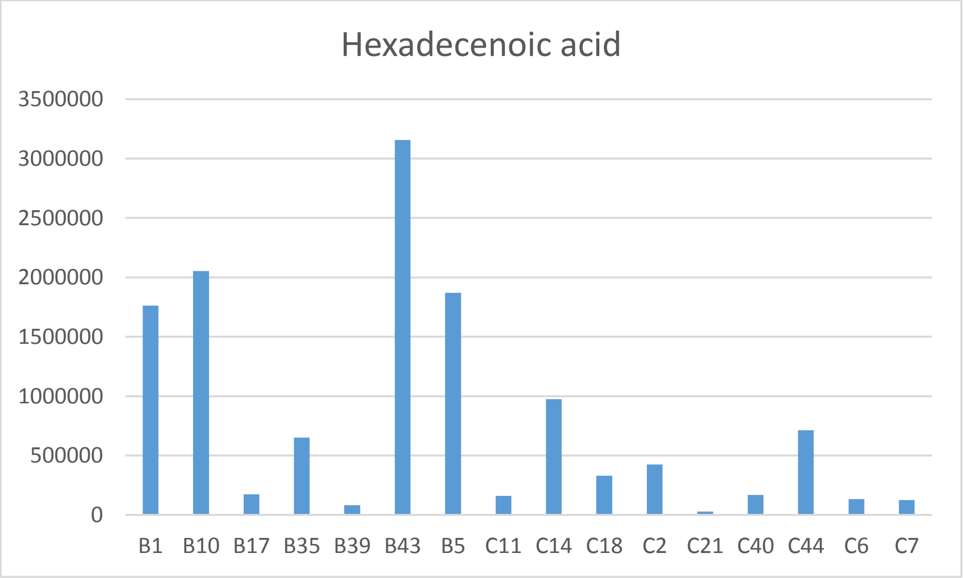

Figure S15 Heptadecenoic acid in pre-80K and post-80K samples

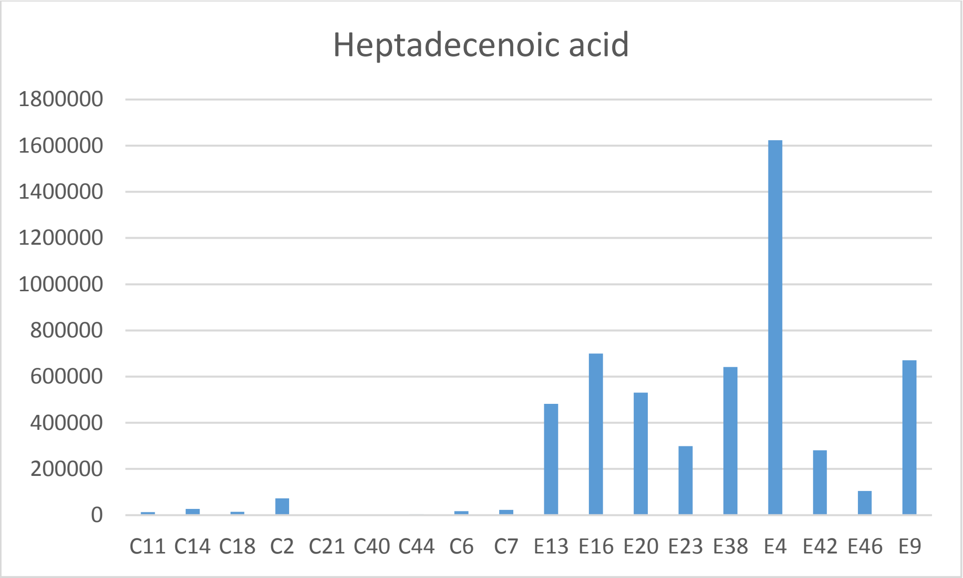

Figure S16 Heptadecenoic acid in baseline and pre-80K samples

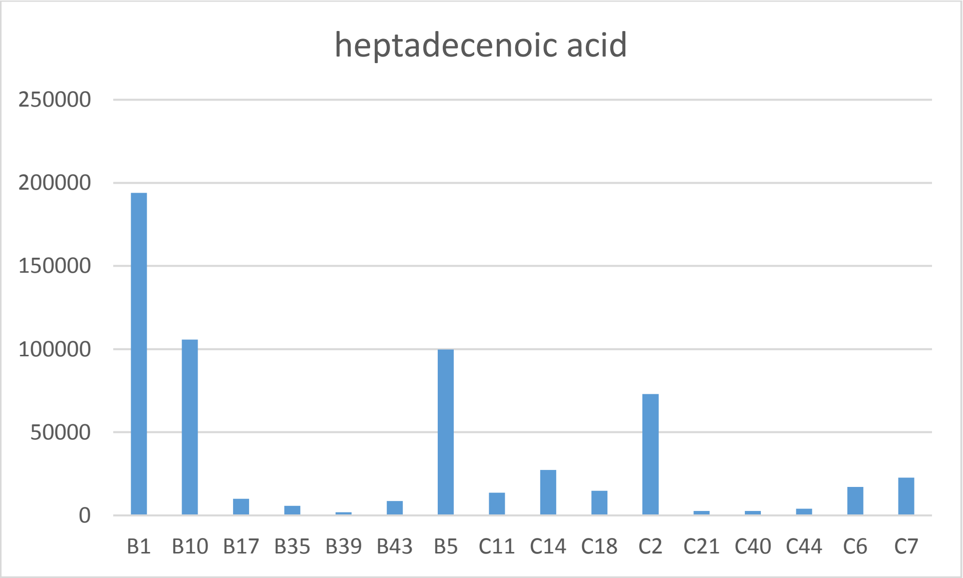

Figure S17 Octatetraenoic acid in pre-80K and post-80K samples

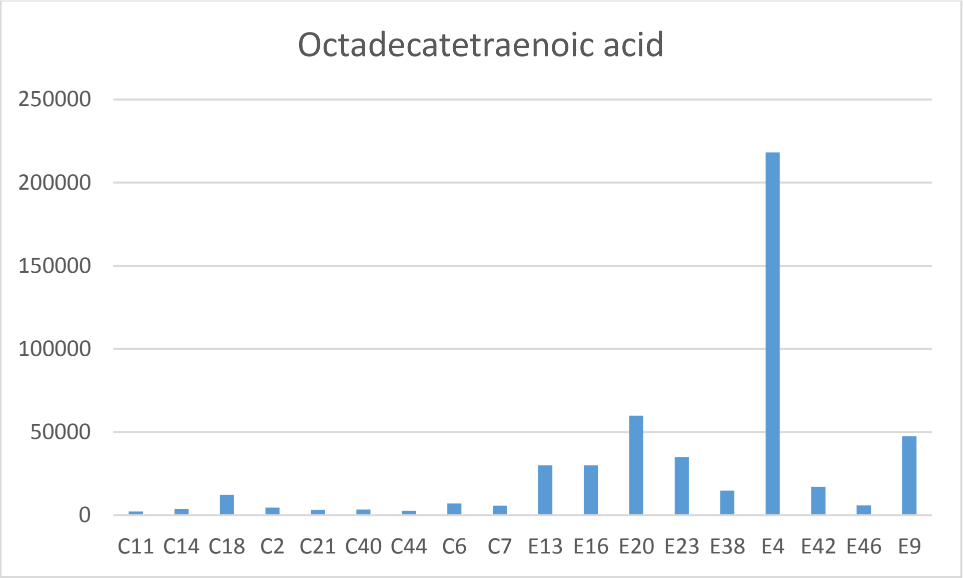

Figure S18 Octatetraenoic acid in baseline and pre-80K samples

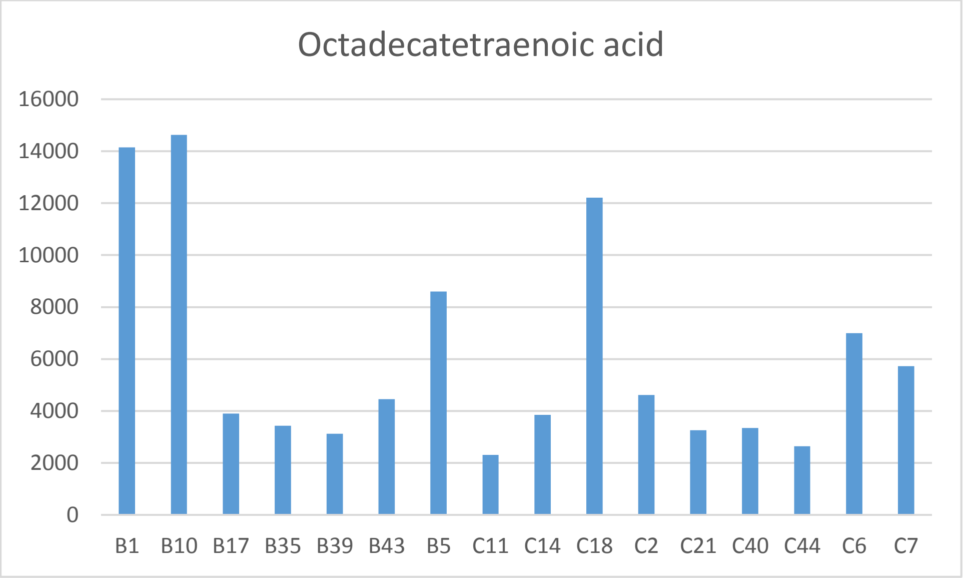

Figure S19 Linoleic acid in pre-80K and post-80K samples

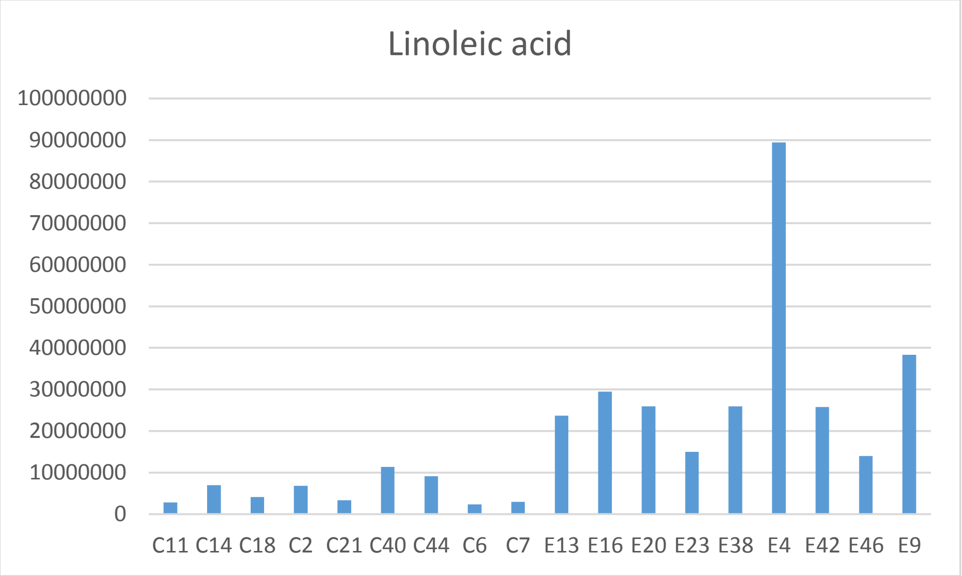

Figure S20 Linoleic acid in baseline and pre-80K samples

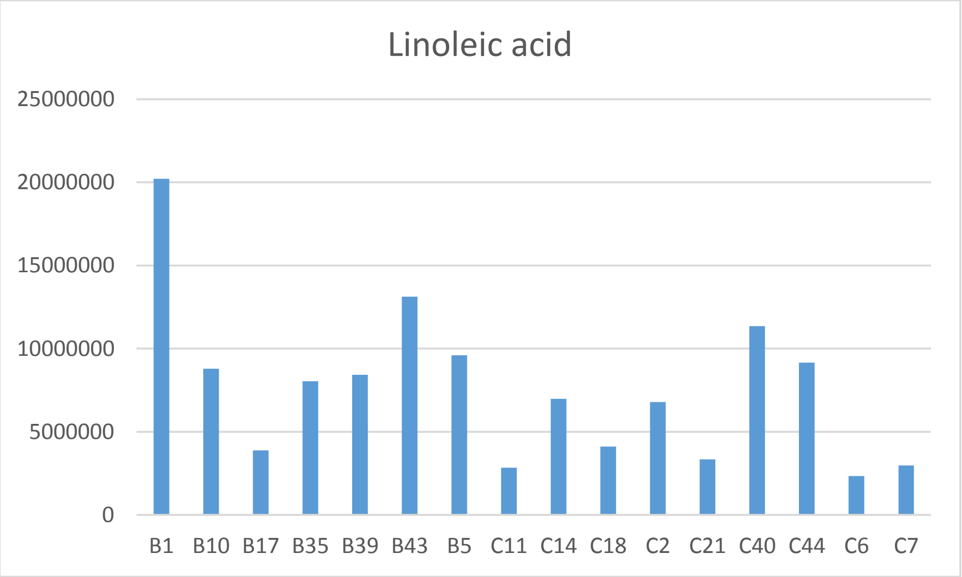

Figure S21 Oleic acid in pre-80K and post-80K samples

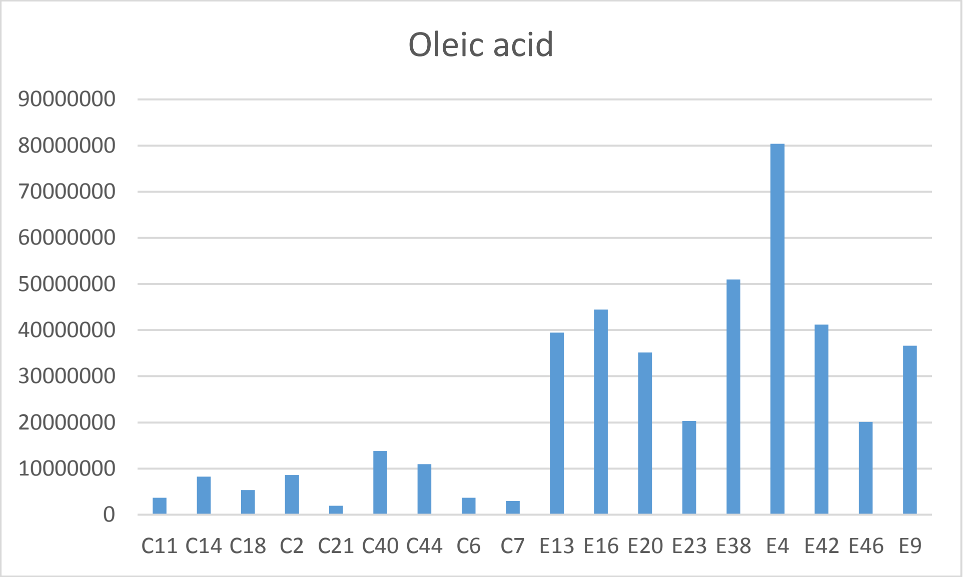

Figure S22 Oleic acid in baseline and pre-80K samples

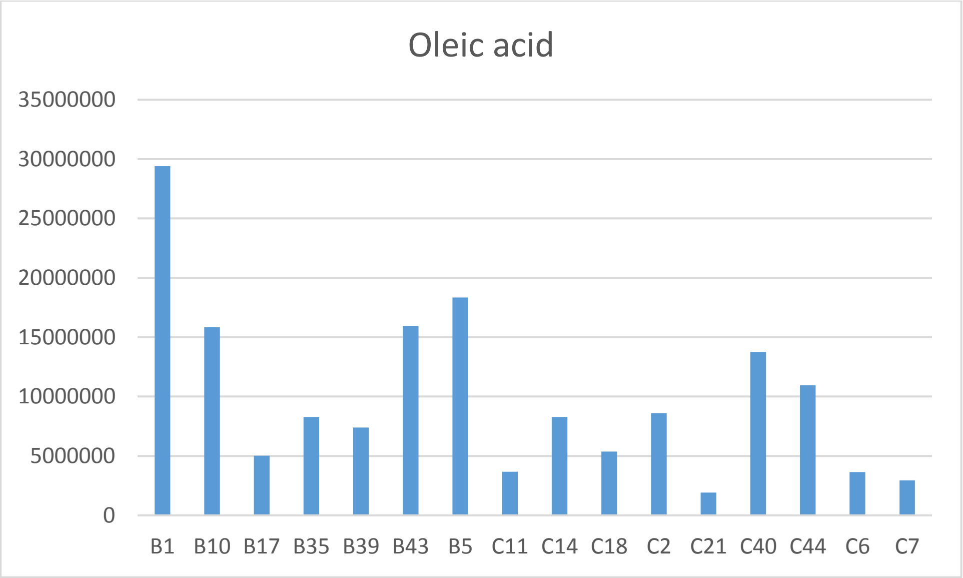

Figure S23 Hydroxyoctadecadienoic acid in pre-80K and post-80K samples

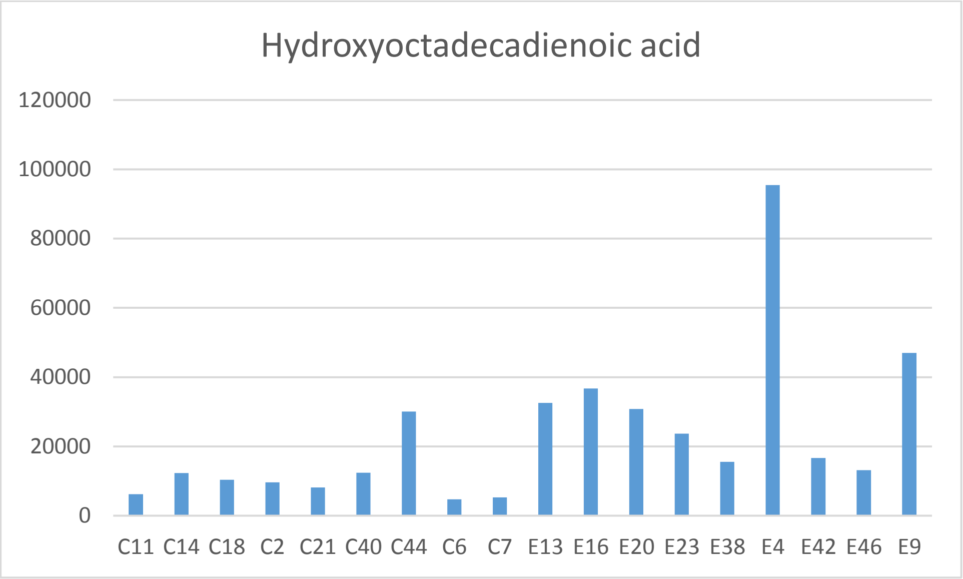

Figure S24 Hydroxyoctadecadienoic acid in baseline and pre-80K samples

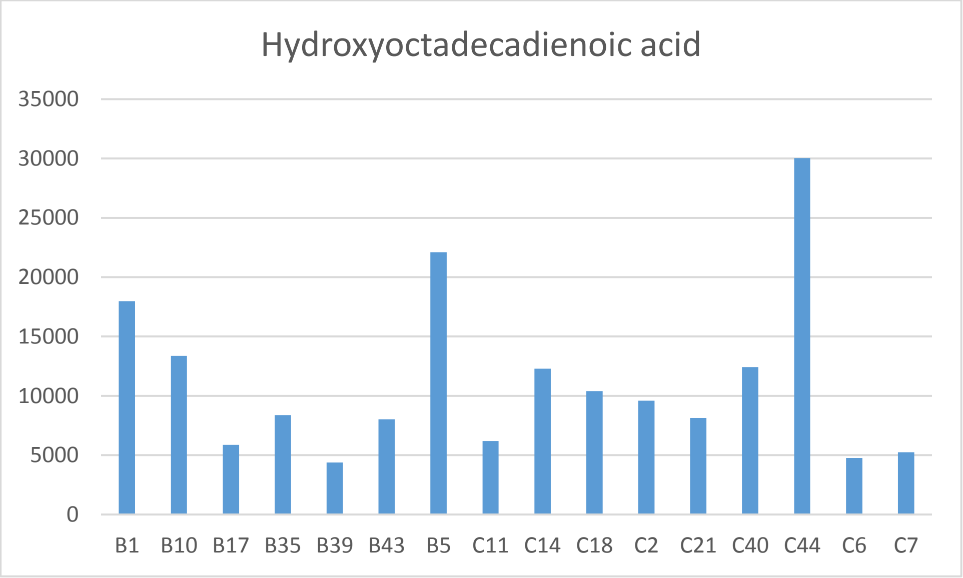

Figure S25 Hydroxyoctadecadenoic acid in pre-80K and post-80K samples

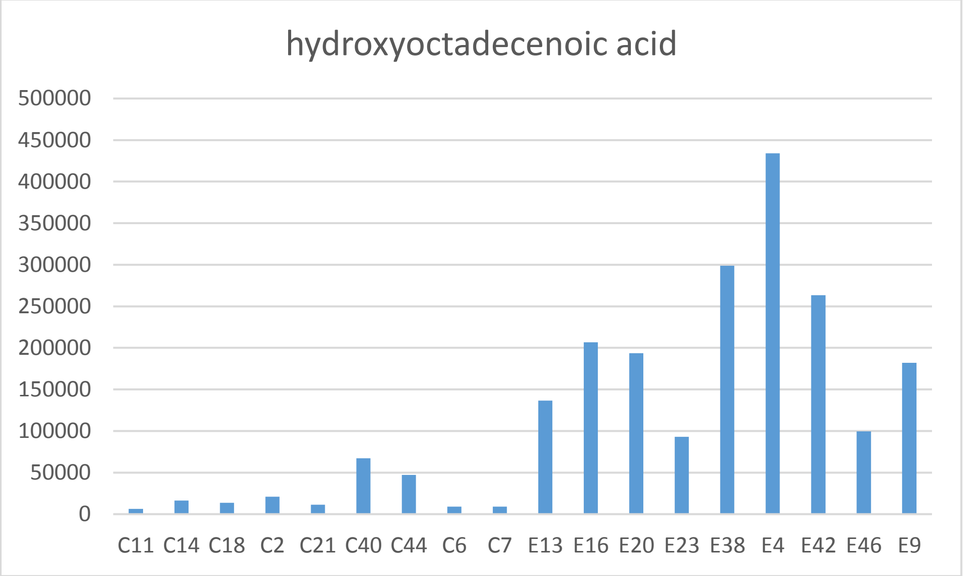

Figure S26 Hydroxyoctadecenoic acid in baseline and pre-80K samples

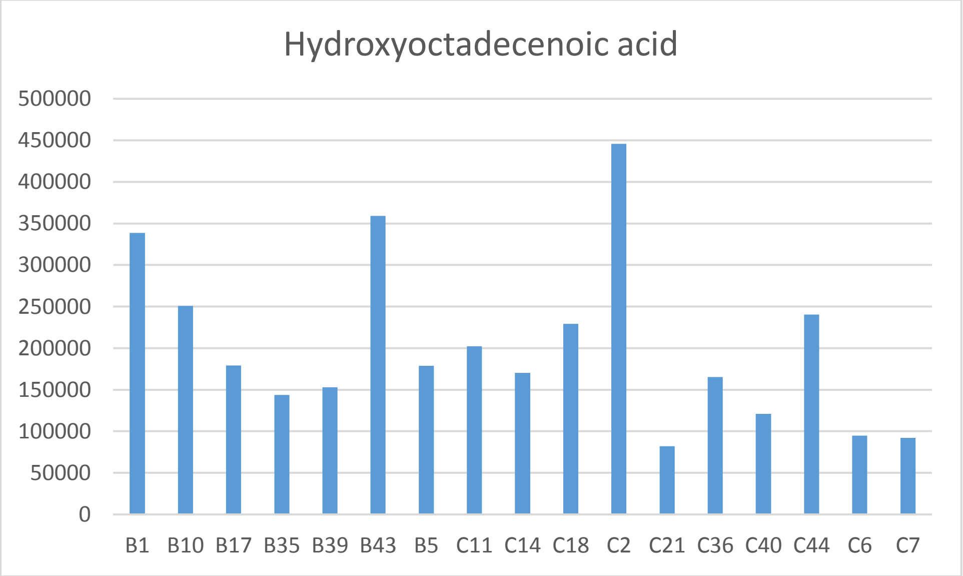

Figure S27 Docasahexaenoic acid in pre-80K and post-80K samples

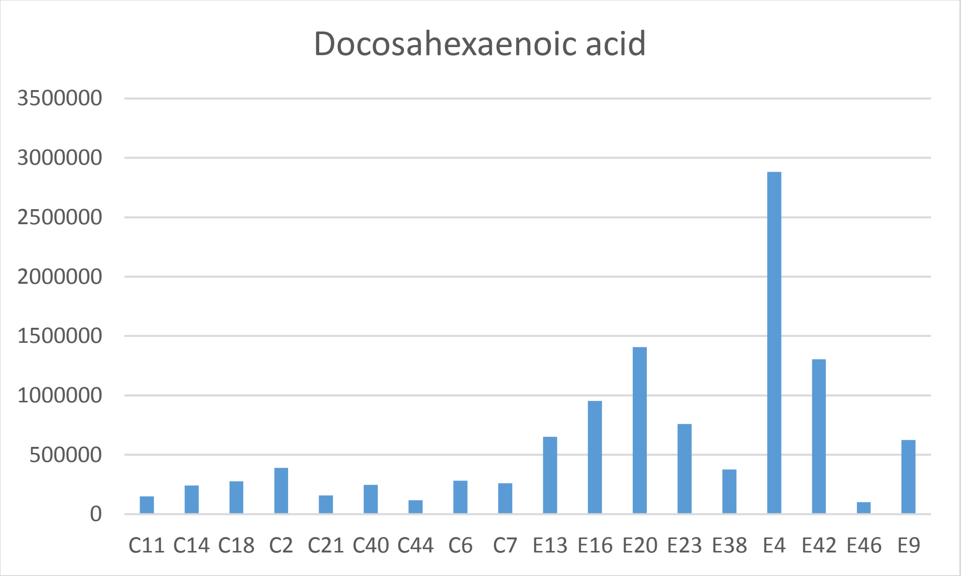

Figure S28 Docosahexaenoic acid in baseline and pre-80K samples

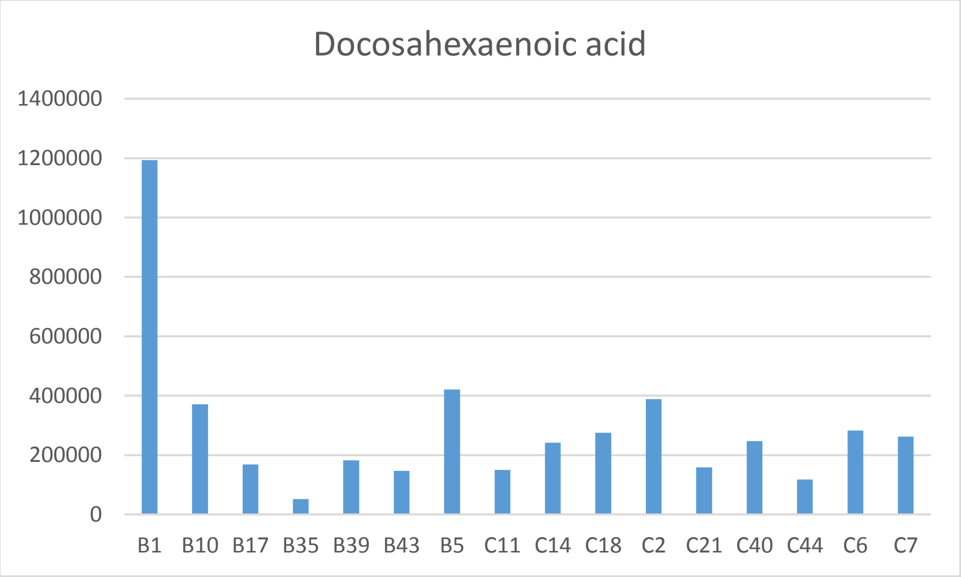

Figure S29 Docasapentaenoic acid in pre-80K and post-80K samples

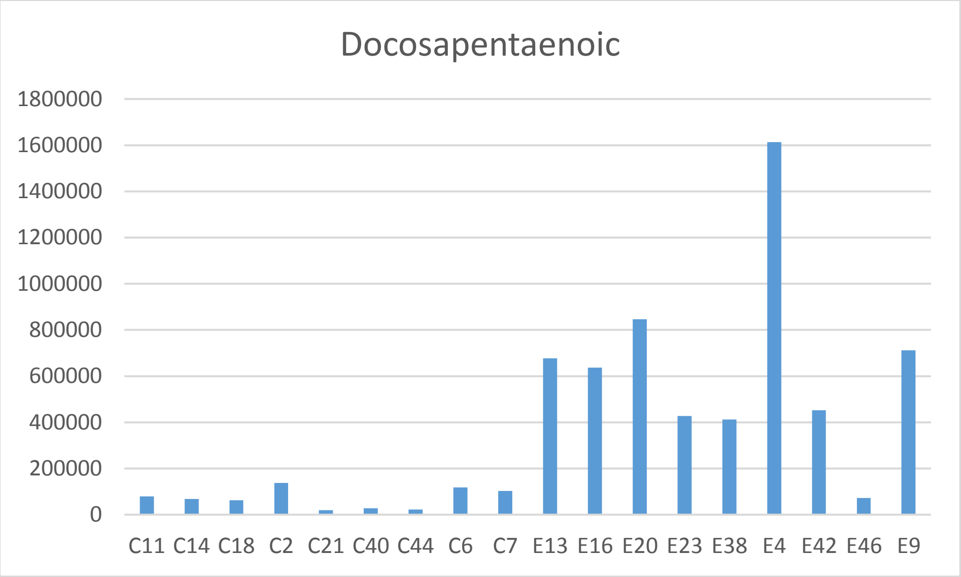

Figure S30 Docasapentaenoic acid in baseline and pre-80K samples

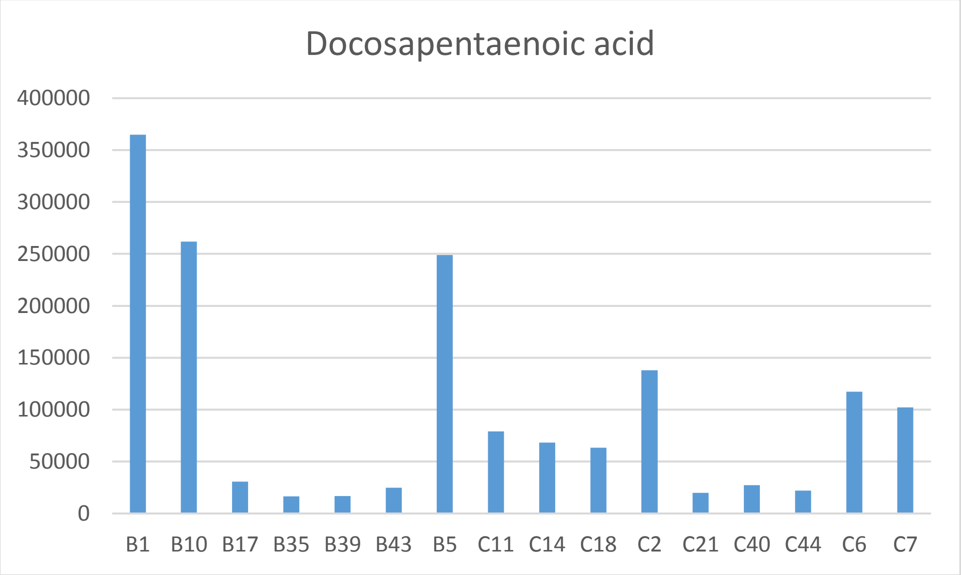

Figure S31 Docasatetraenoic acid in pre-80K and post-80K samples

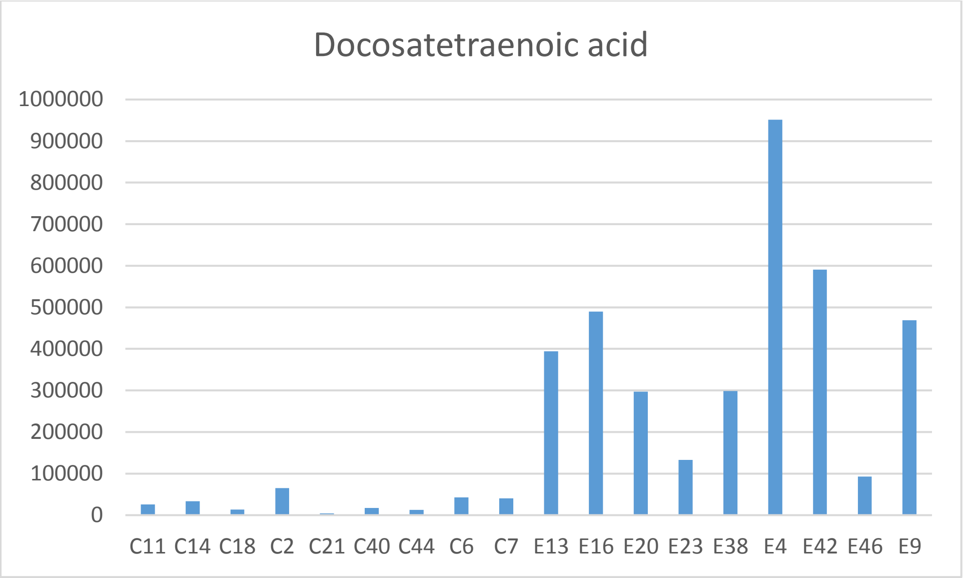

Figure S32 Docosatetraenoic acid in baseline and pre-80K samples

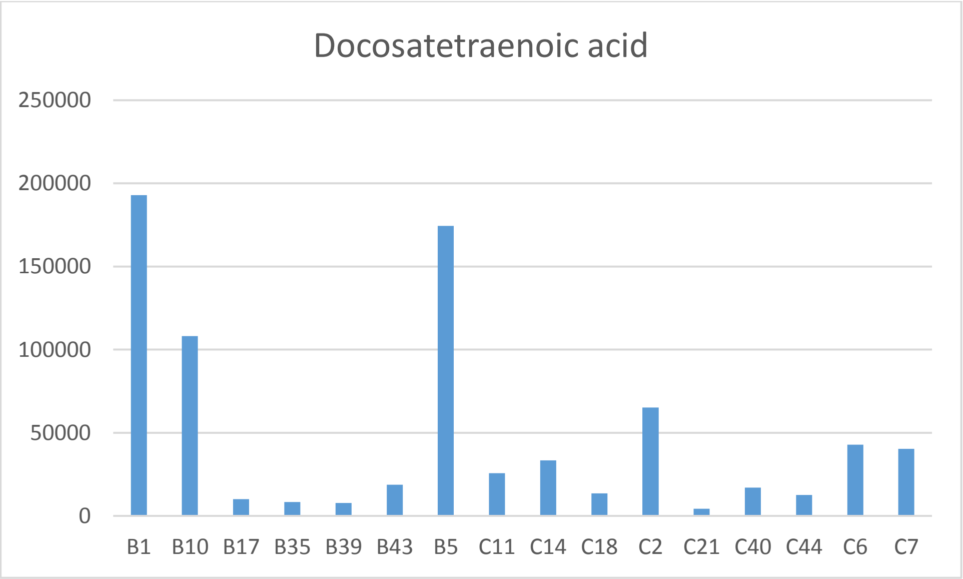

Figure S33 Dihydroxyoctadecenoic acid

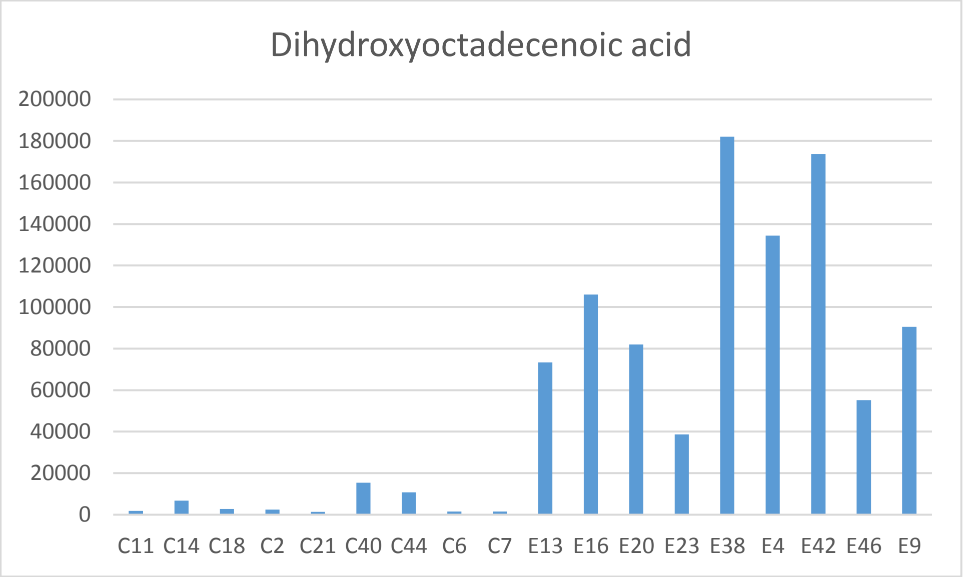

Figure S34 Dihydroxyoctadecenoic acid in baseline and pre-80K samples

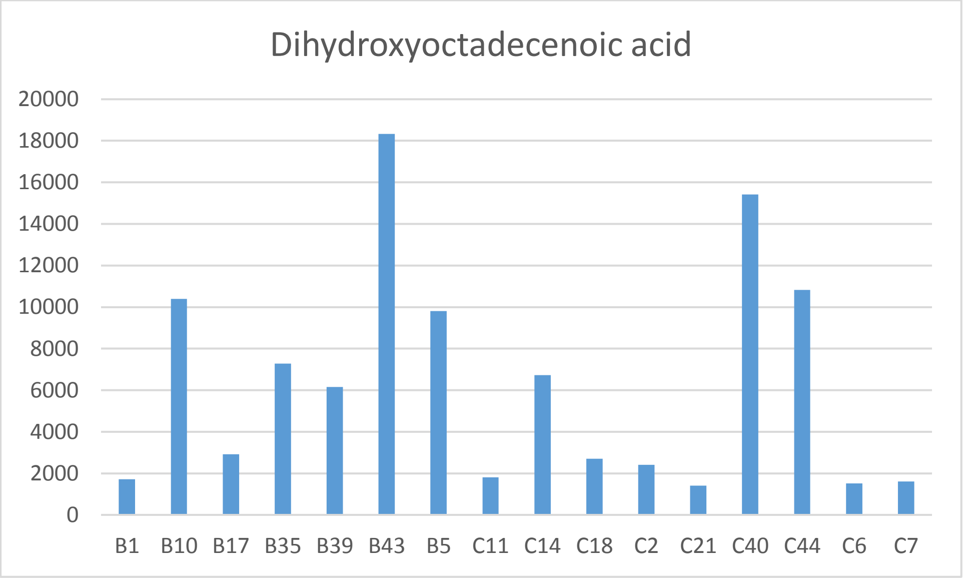

Figure S35 Acetylcarnitine in pre-80K and post-80K samples

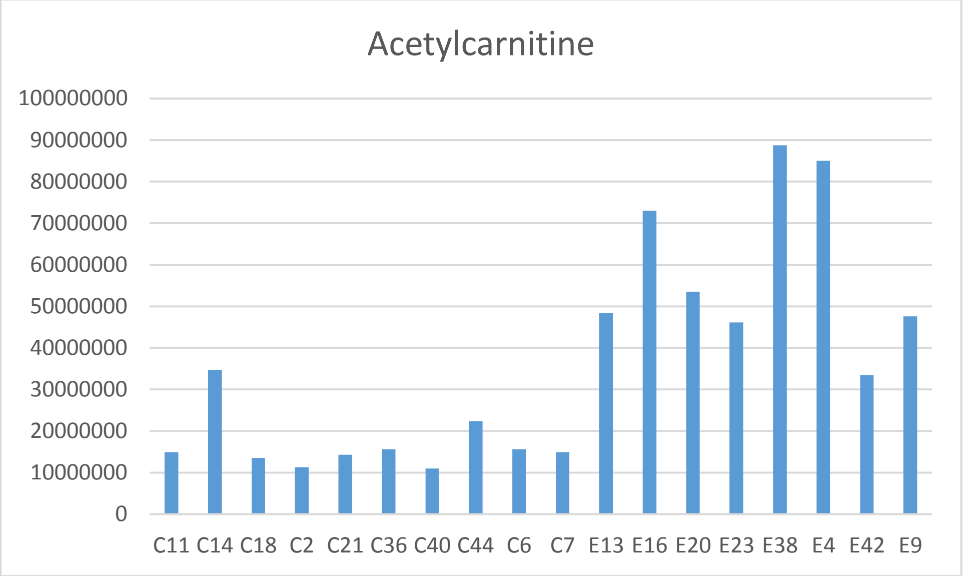

Figure S36 Acetylcarnitine in baseline and pre-80K samples

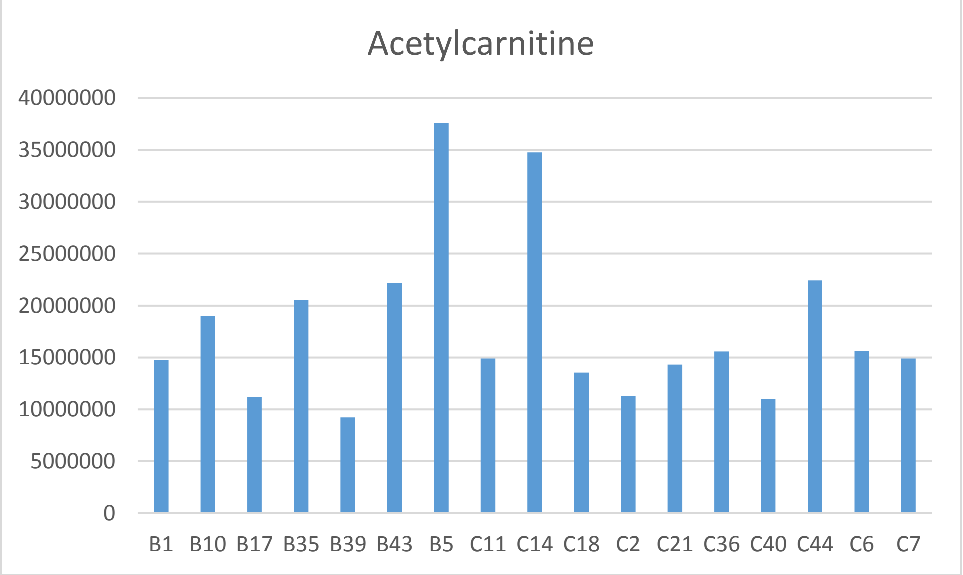

Figure S37 Octenoylcarnitine in pre-80K and post-80K samples

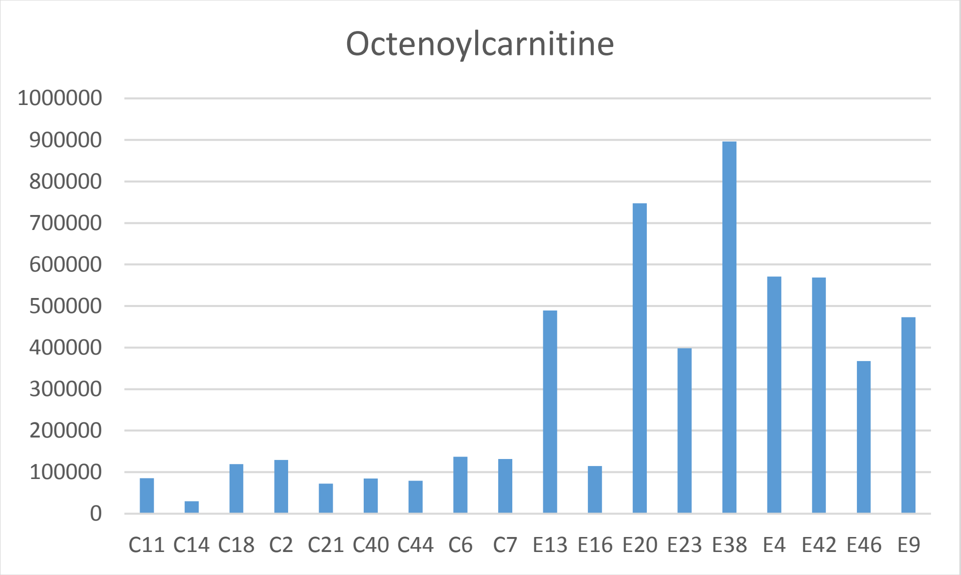

Figure S38 Octenoylcarnitine in baseline and pre-80K samples

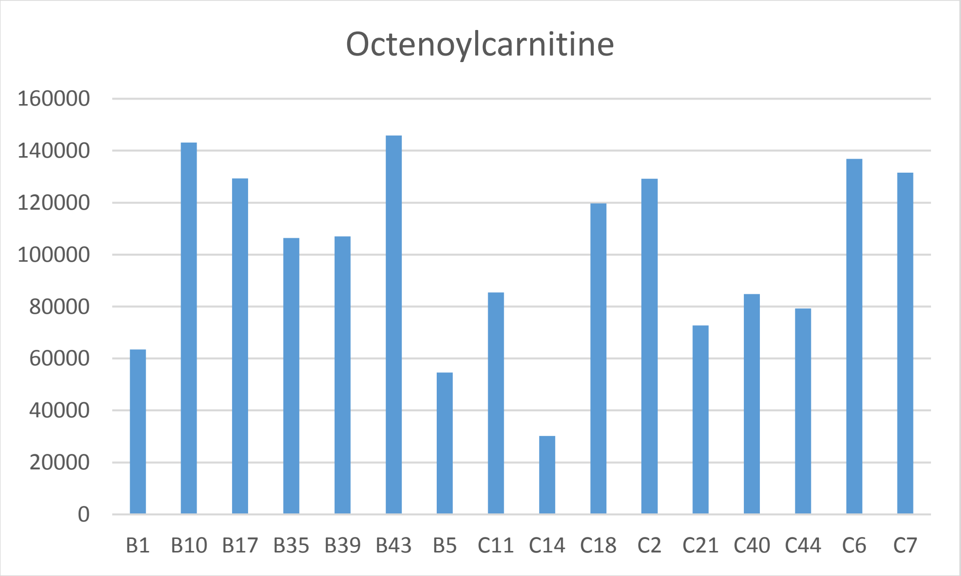

Figure S39 Octanoylcarnitine in pre-80K and post-80K samples

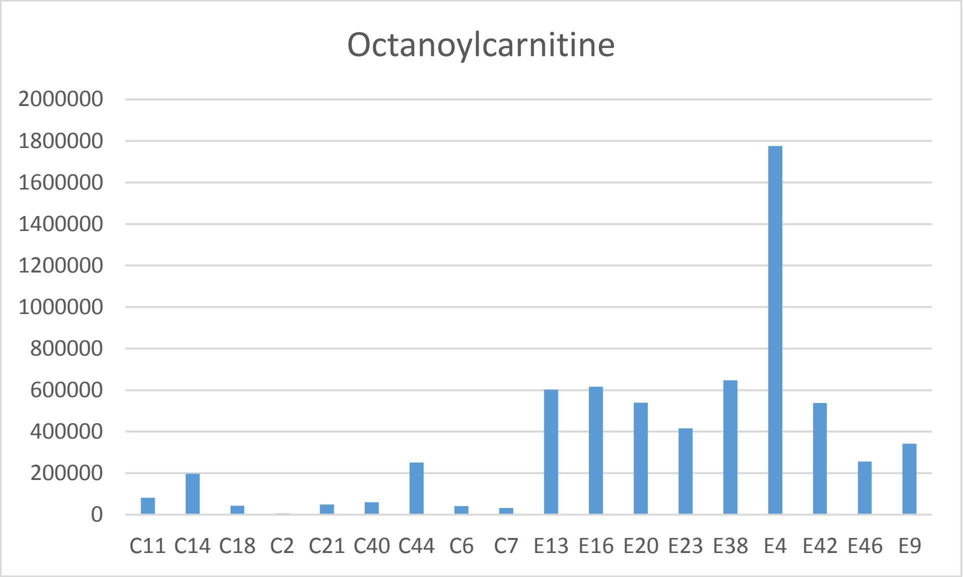

Figure S40 Octanoylcarnitine in baseline and pre-80K samples

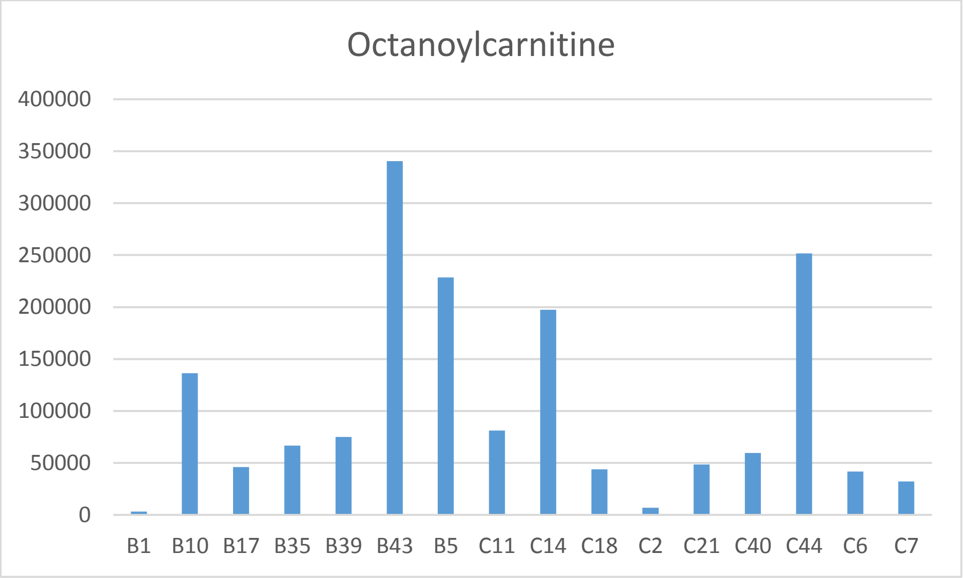

Figure S41 Nonanoylcarnitine in pre-80K and post-80K samples

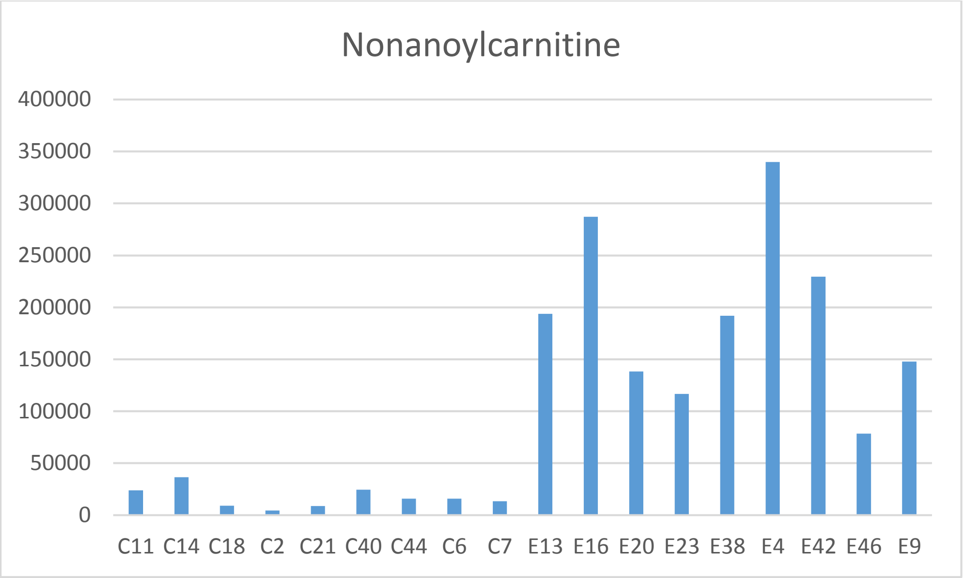

Figure S42 Nonanoylcarnitine in baseline and pre-80K samples

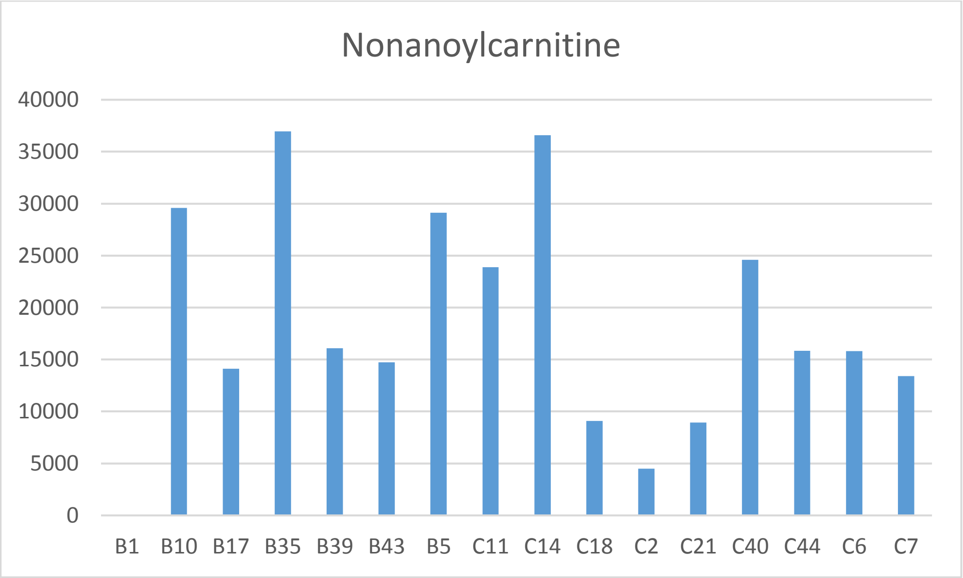

Figure S43 Decadienoylcarnitine in pre-80K and post-80K samples

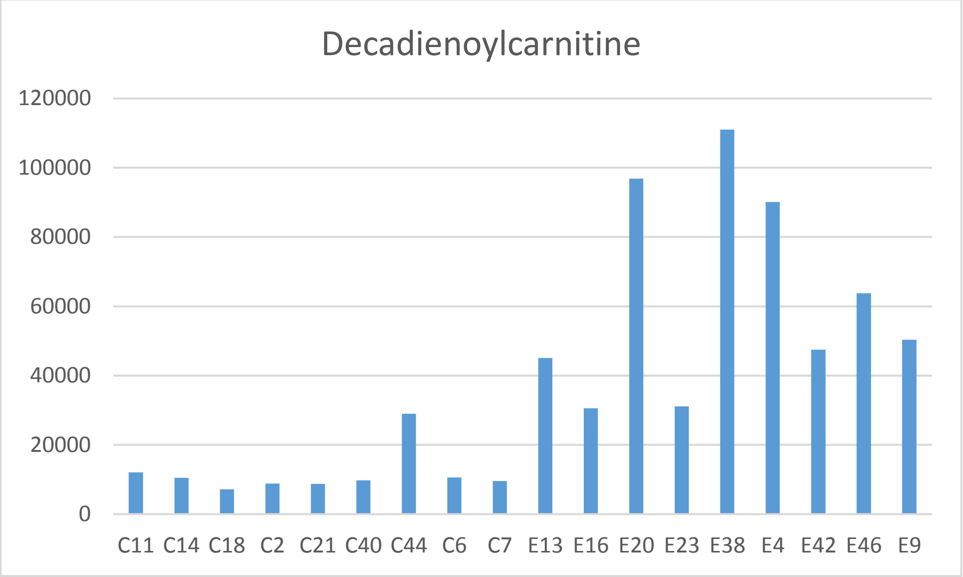

Figure S44 Decadienoylcarnitine in baseline and pre-80K samples

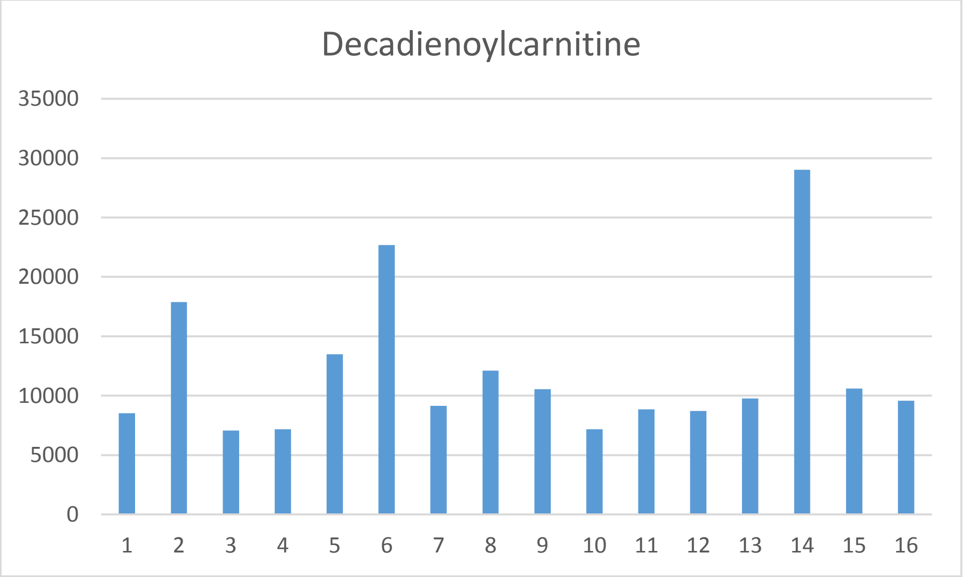

Figure S45 Decenoylcarnitine in pre-80K and post-80K samples

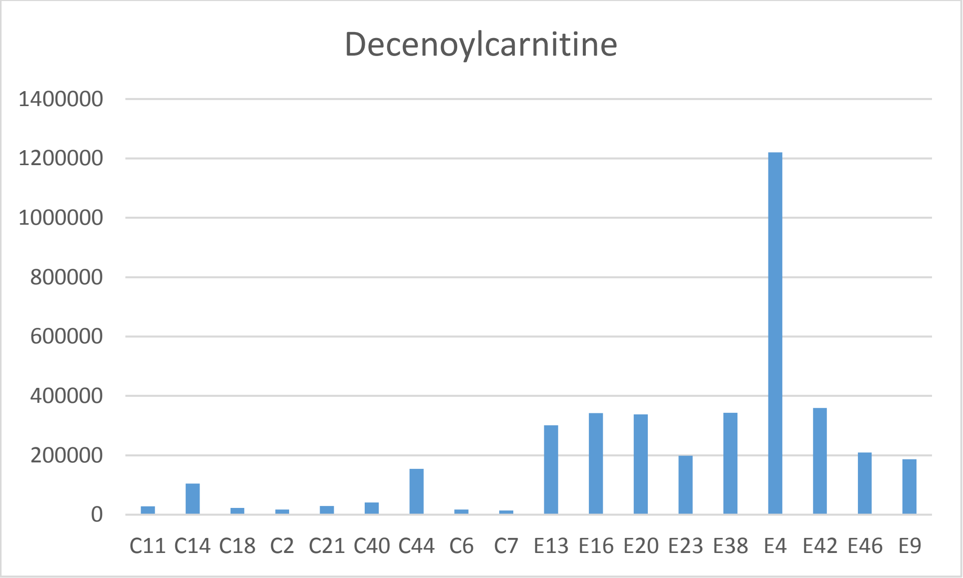

Figure S46 Decenoylcarnitine in baseline and pre-80K samples

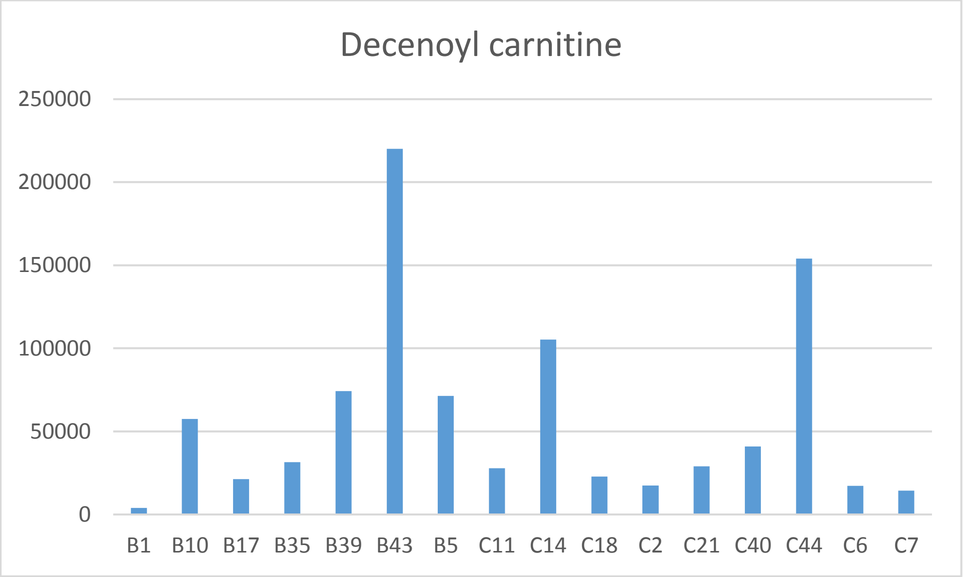

Figure S47 Decanoylcarnitine in pre-80K and post-80K samples

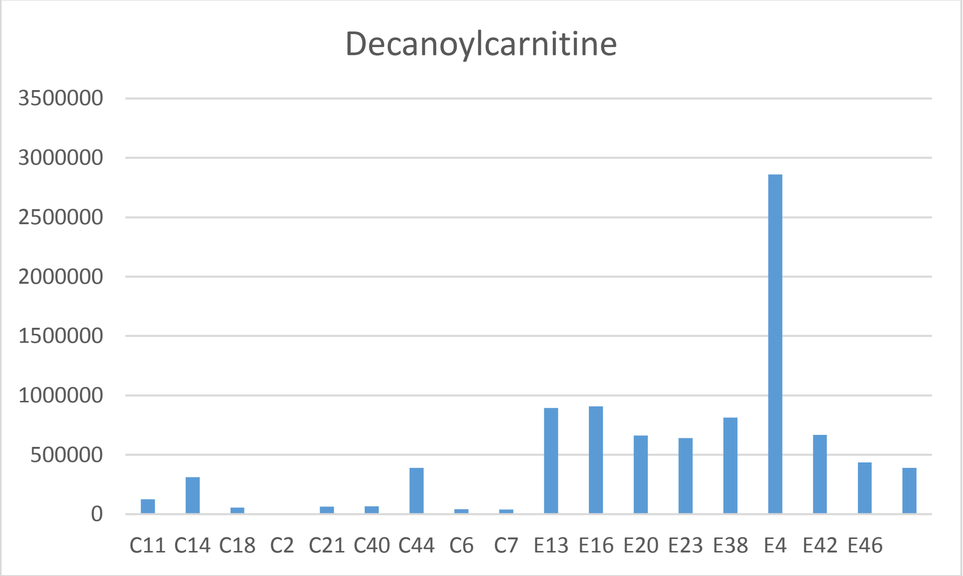

Figure S48 Decanoylcarnitine in baseline and pre-80K samples

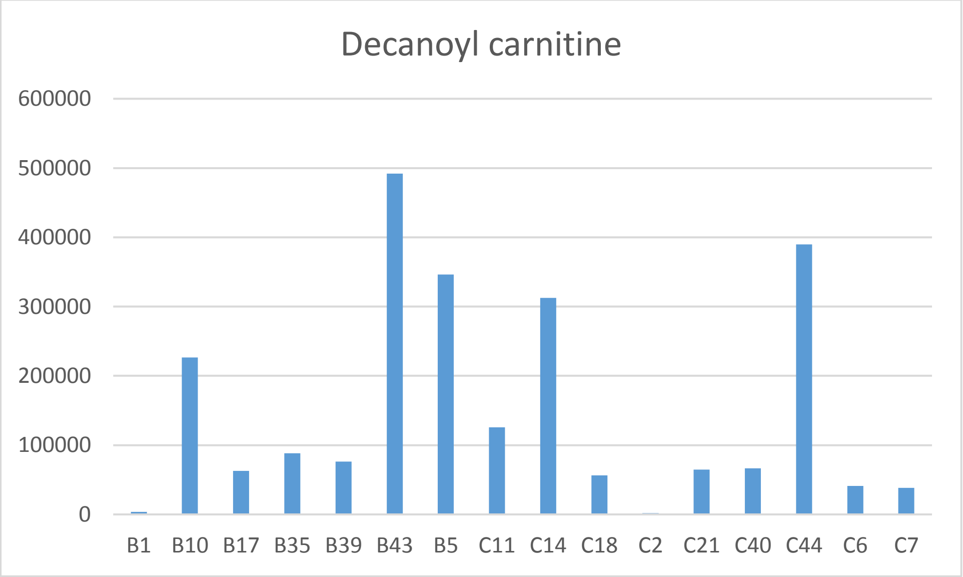

Figure S49 Dodecanoylcarnitine in pre-80K and post-80K samples

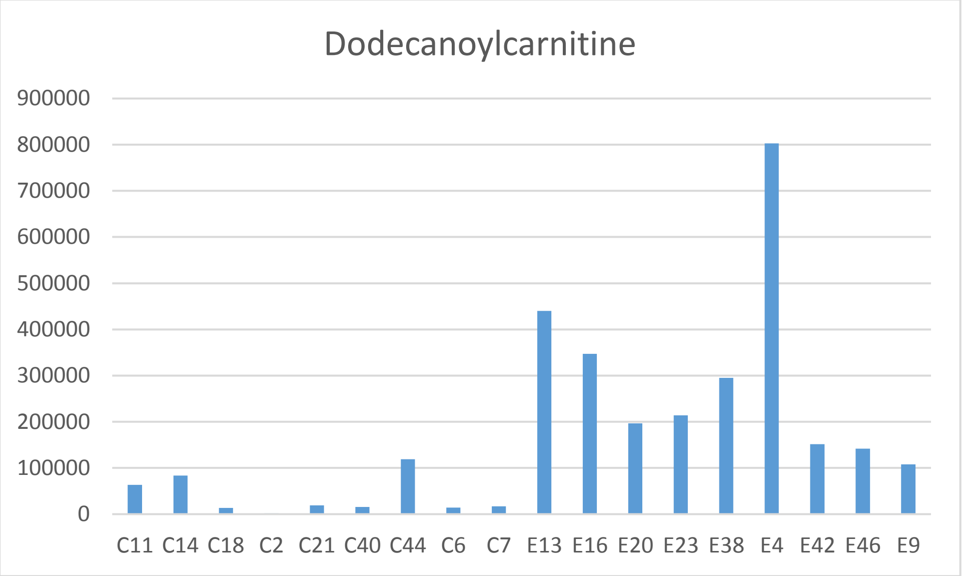

Figure S50 Dodecanoylcarnitine in baseline and pre-80K samples

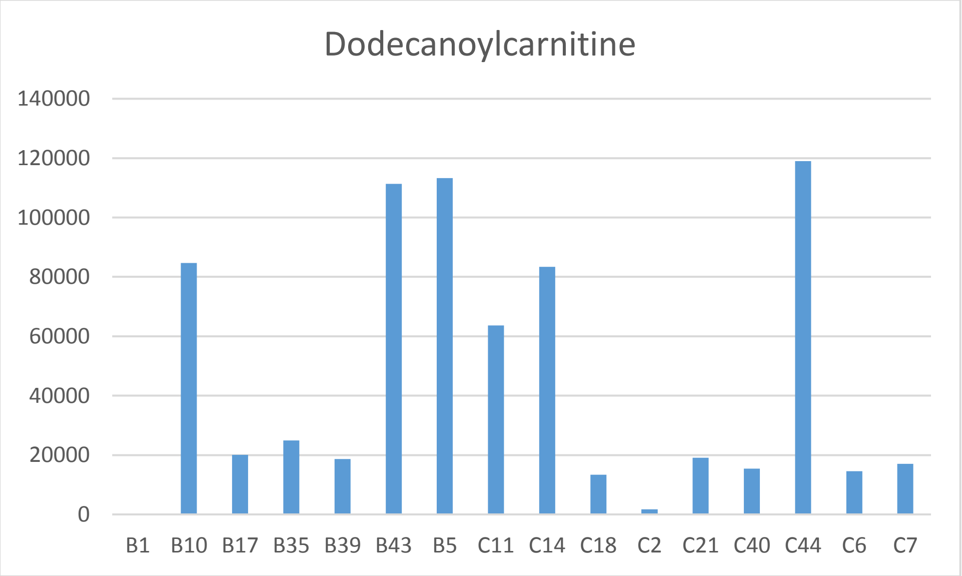

Figure S51 Tetradecanoylcarnitine in pre-80K and post-80K samples.

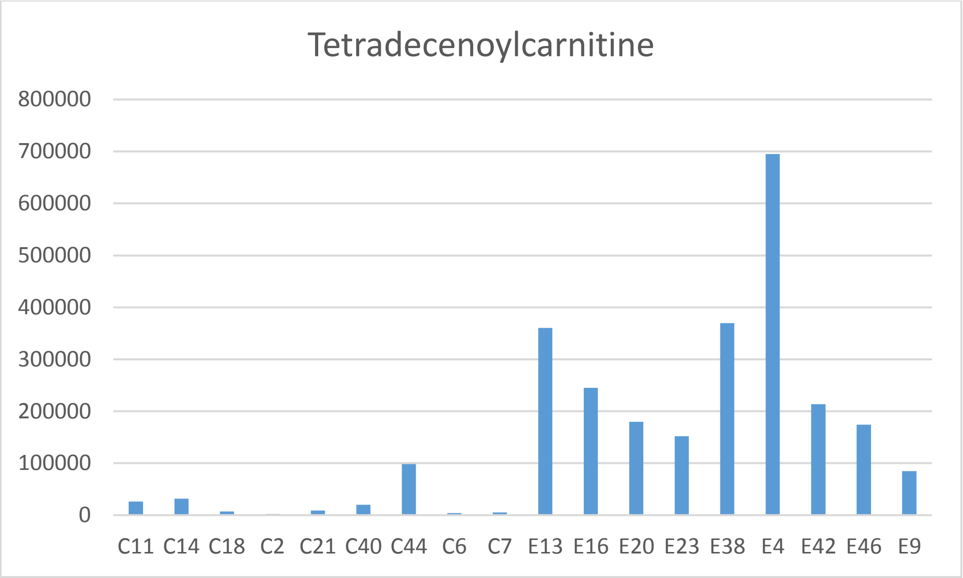

Figure S52 Tetradecenoylcarnitine in baseline and pre-80K samples

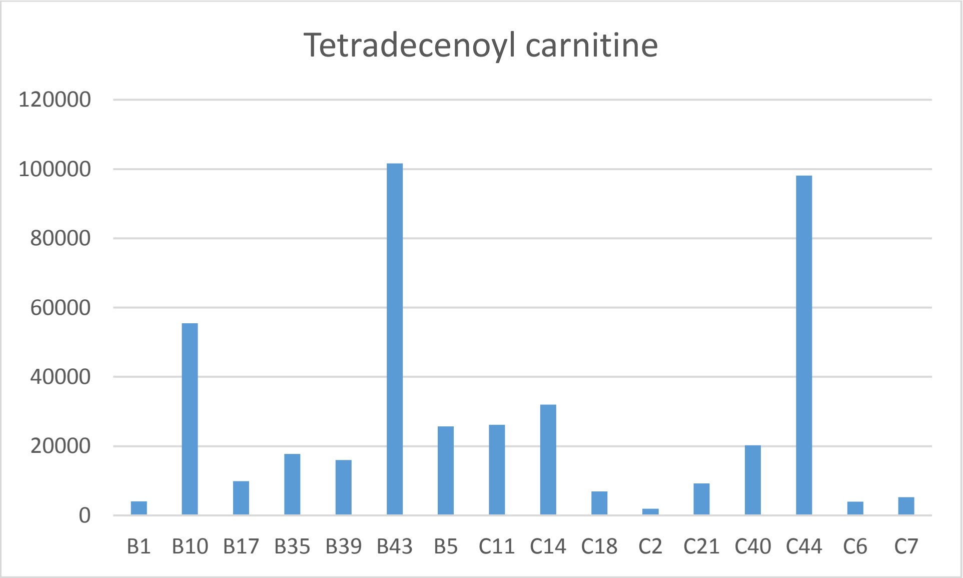

Figure S53 Palmitoylcarnitine in pre-80K and post-80K samples

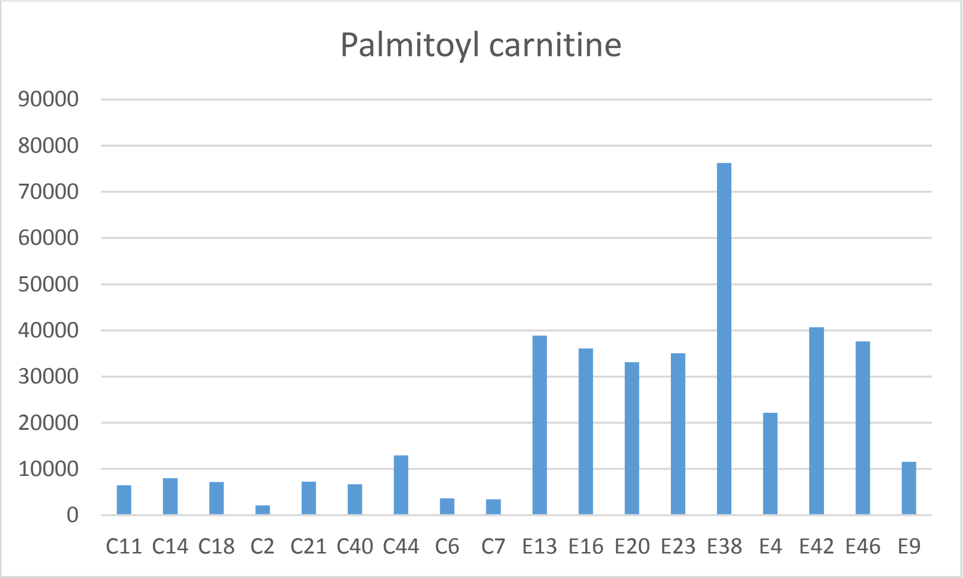

Figure S54 Palmitoylcarnitine in baseline and pre-80K samples

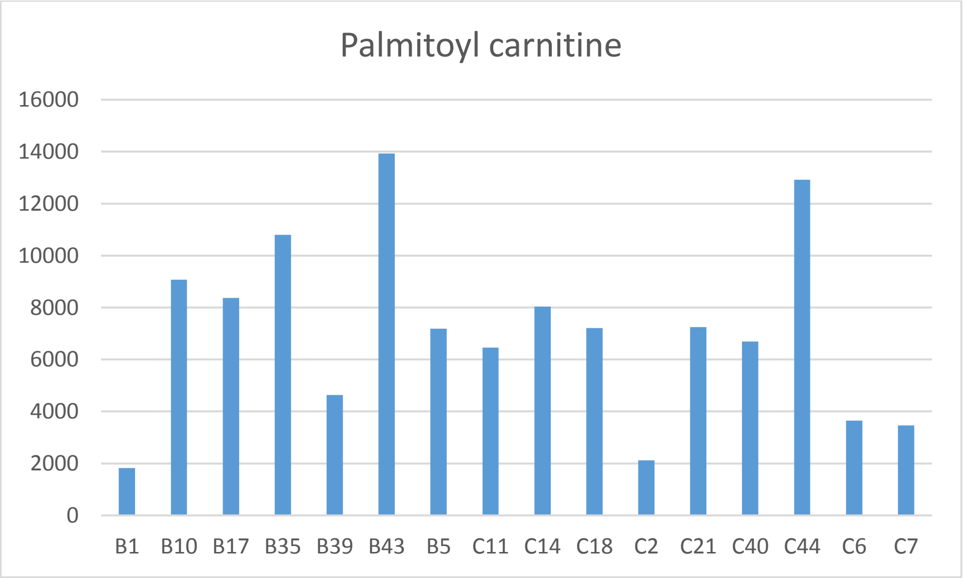

Figure S55 Proline in pre-80K and post-80K samples

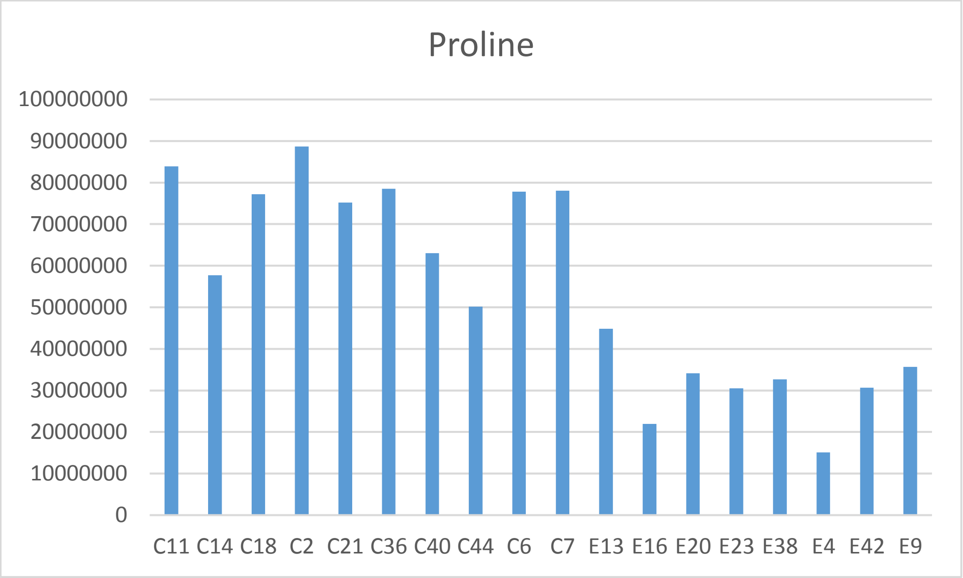

Figure S56 Proline in baseline and pre-80K samples

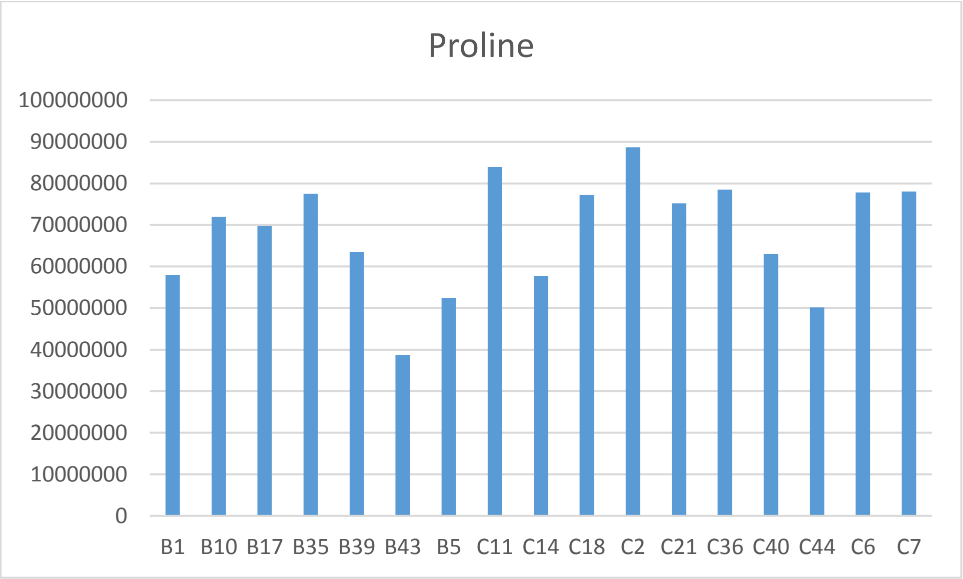

Figure S57 Valine in pre-80K and post-80K samples

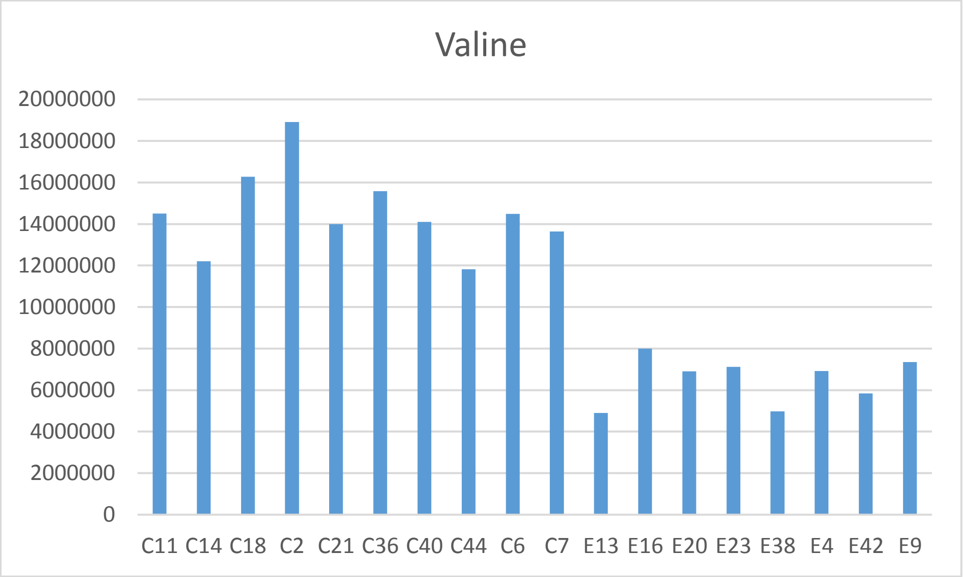

Figure S58 Valine in baseline and pre-80K samples

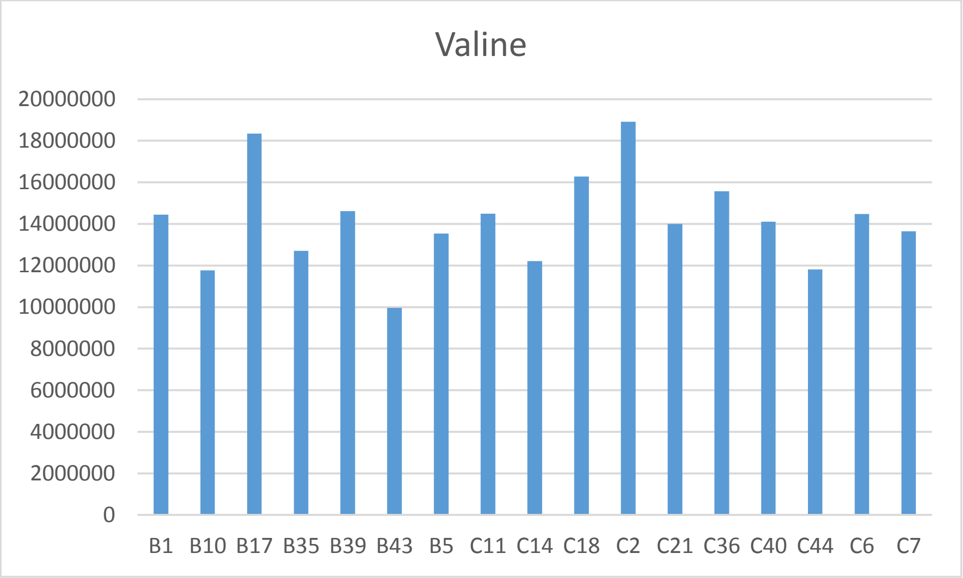

Figure S59 Betaine in pre-80K and post-80K samples

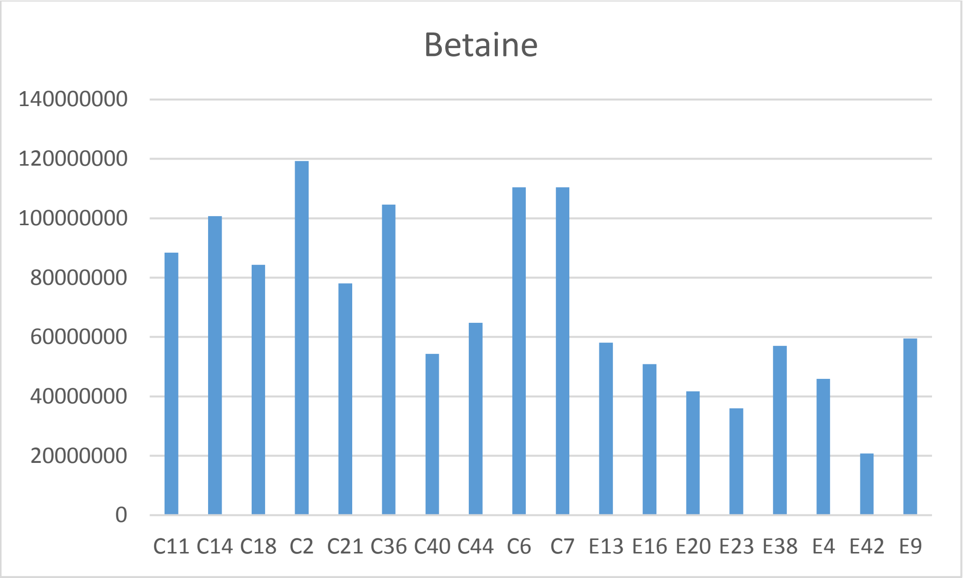

Figure S60 Betaine in baseline and pre-80K samples

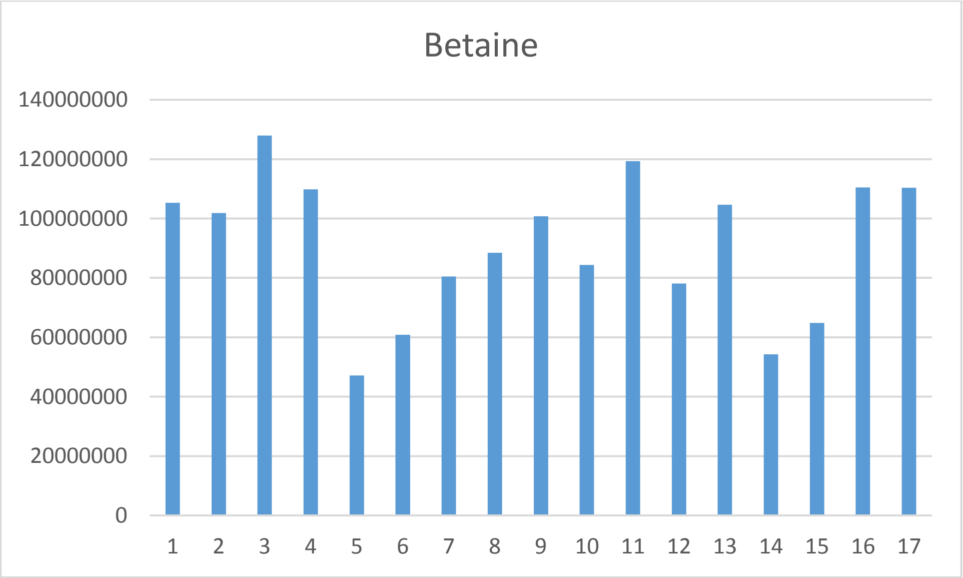

Figure S61 Leucine in pre-80K and post-80K samples

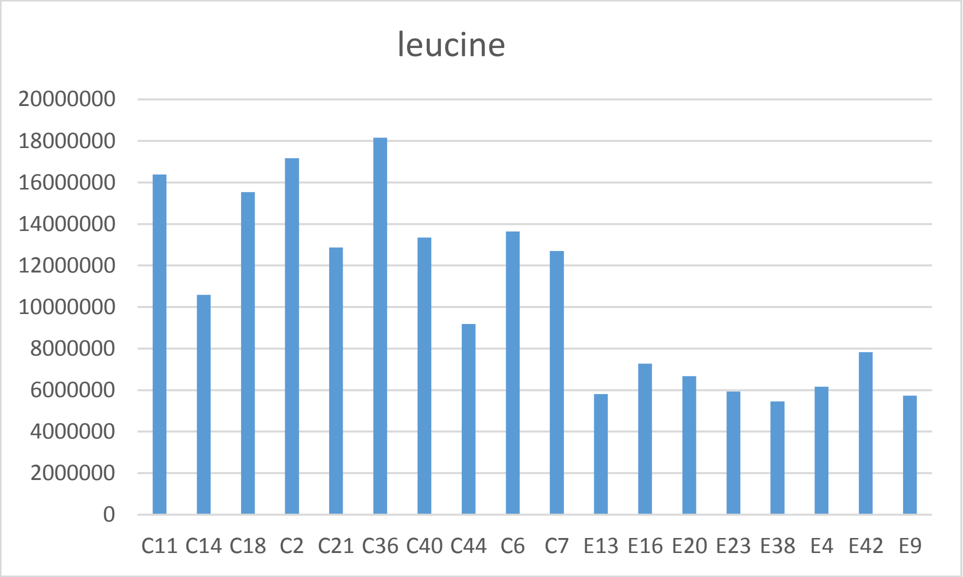

Figure S62 Leucine in baseline and pre-80K samples

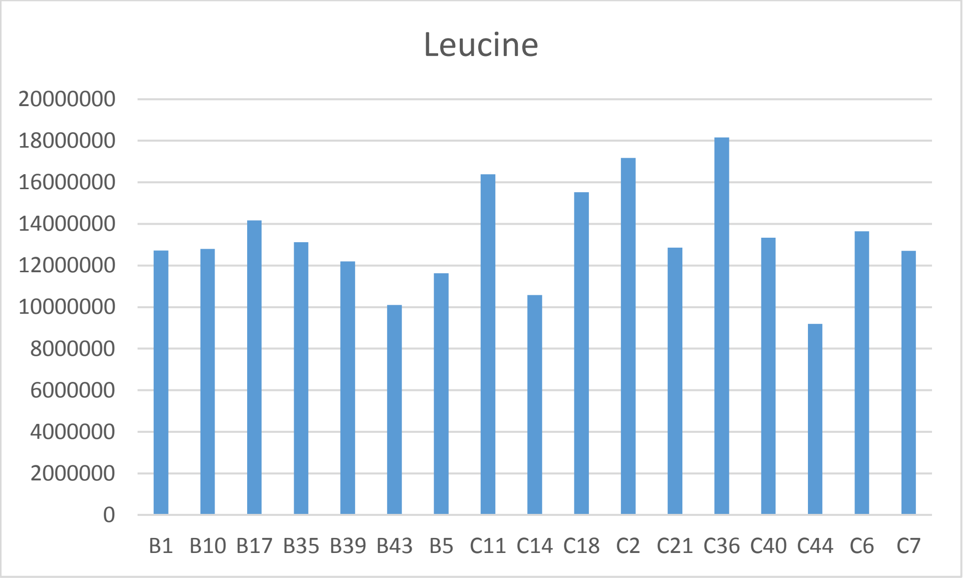

Figure S63 Isoleucine in pre-80K and post-80K samples

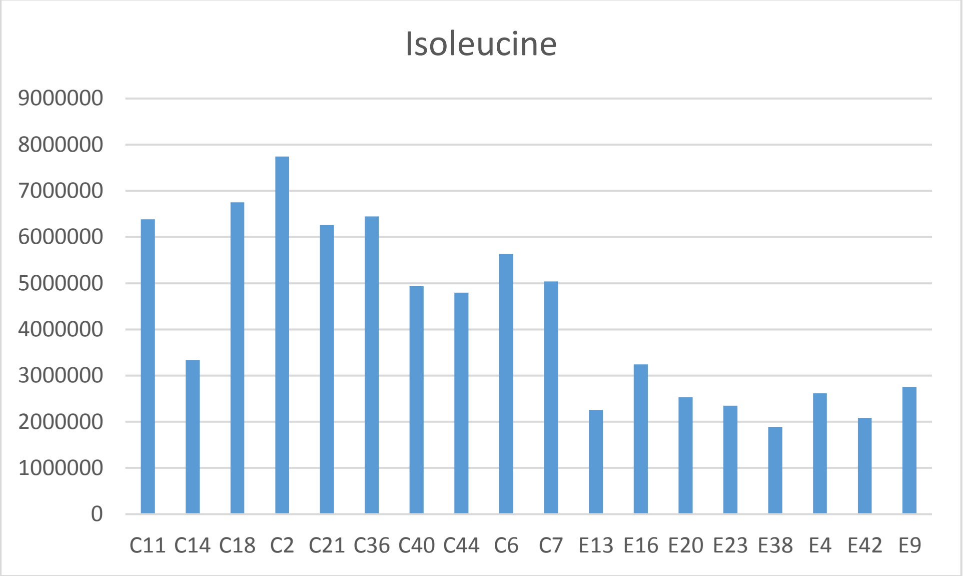

Figure S64 Isoleucine in baseline and pre-80K samples

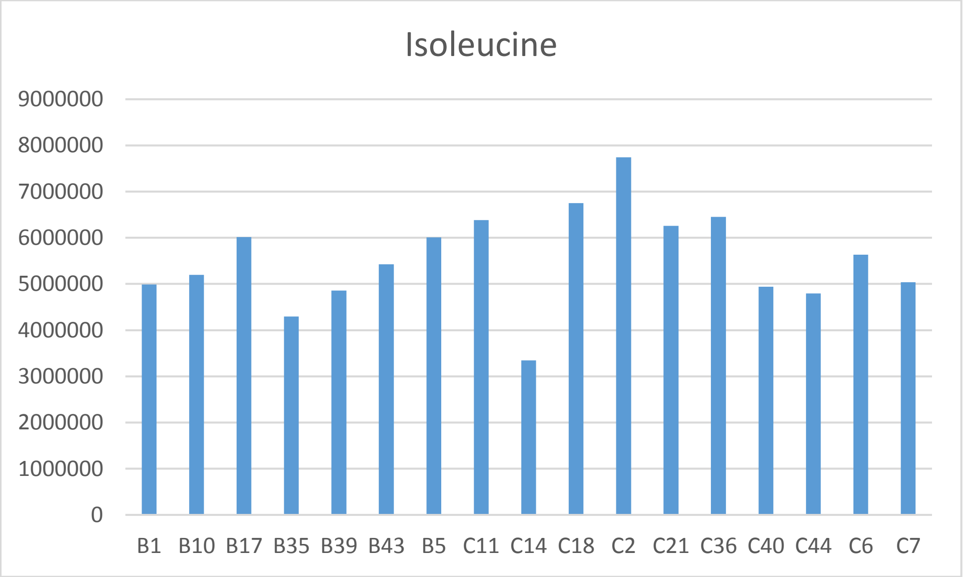

Figure S65 Tryptophan in pre-80K and post-80K samples

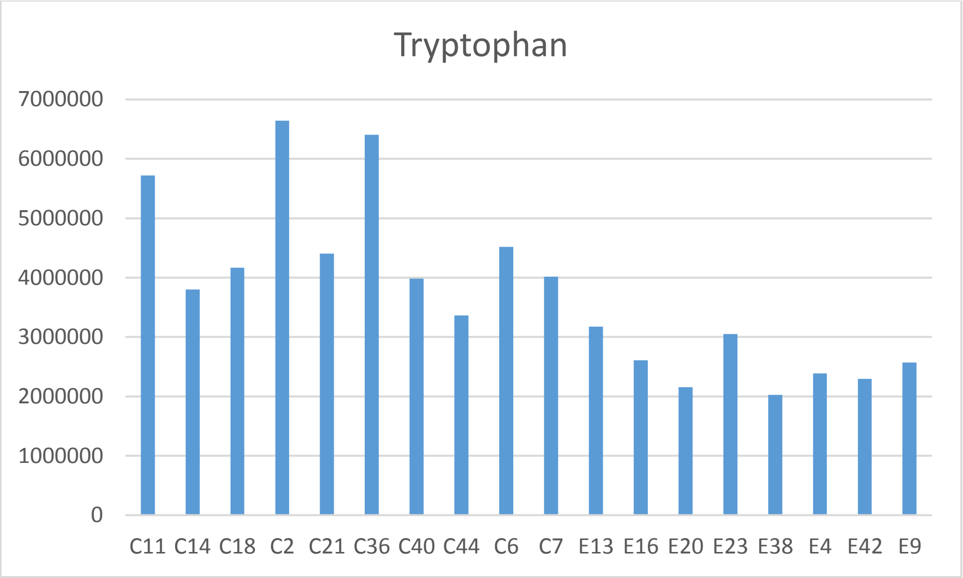

Figure S66 Tryptophan in baseline and pre-80K samples

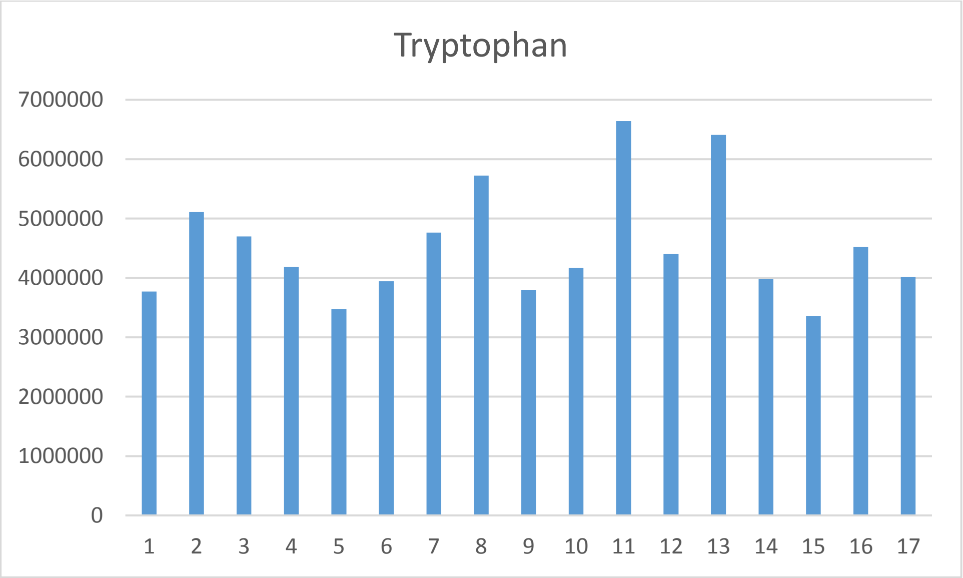

Figure S67 Hydrocortisone in pre-80K and post-80K samples

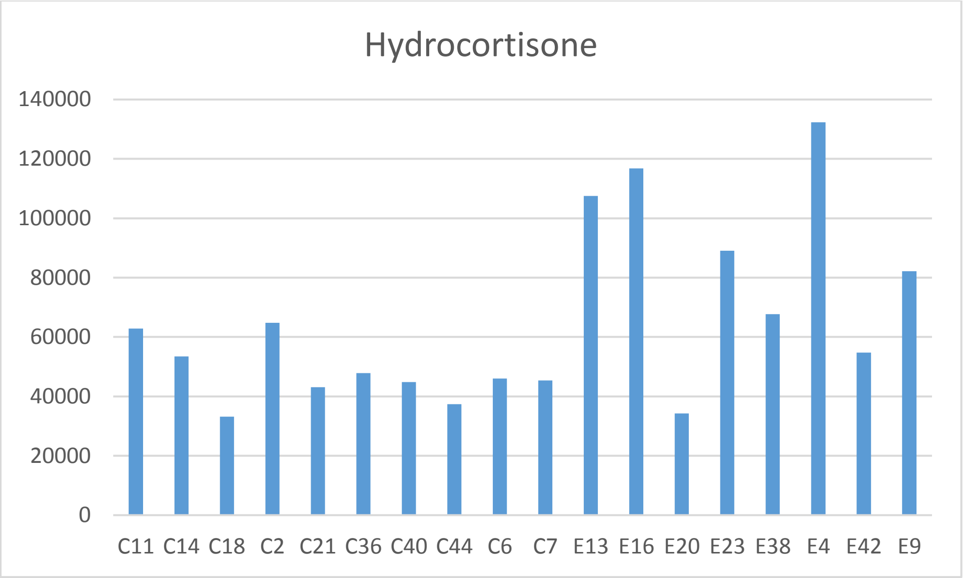

Figure S68 Hydrocortisone in baseline and pre-80K samples

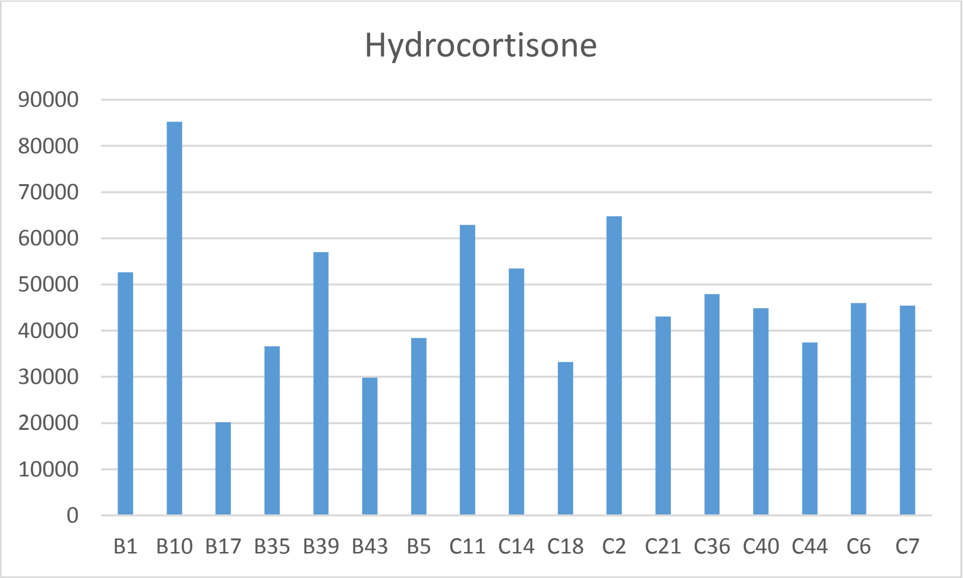

Figure S69 Urocortisone in pre-80K and post-80K samples

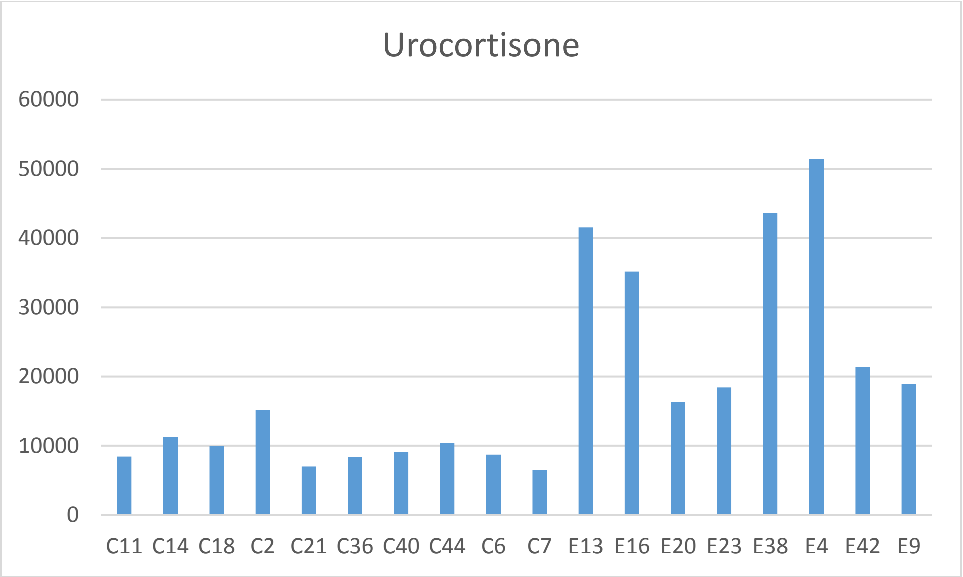

Figure S70 Urocortisone in baseline and pre-80K samples

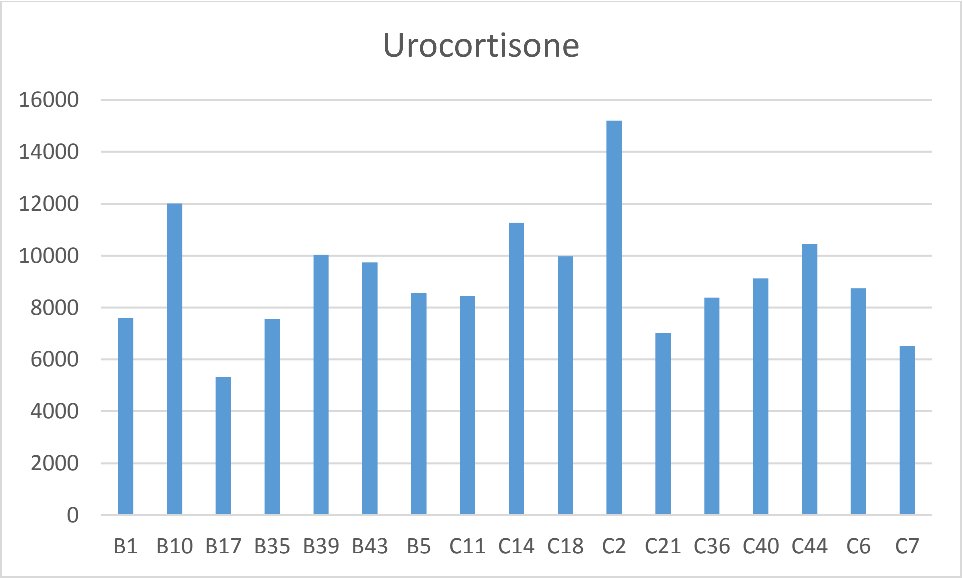

Figure S71 Hypoxanthine in pre-80K and post-80K samples

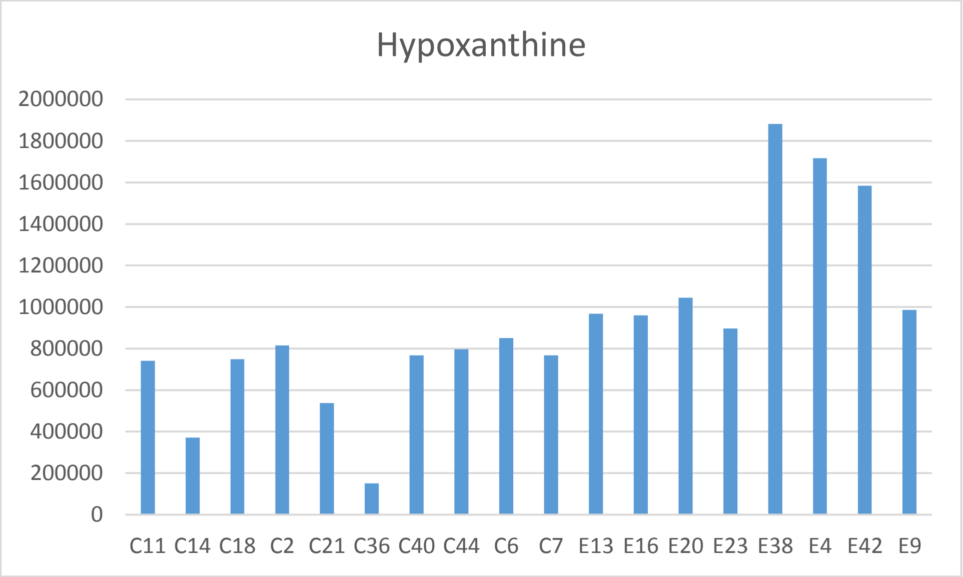

Figure S72 Hypoxanthine in baseline and pre-80K samples

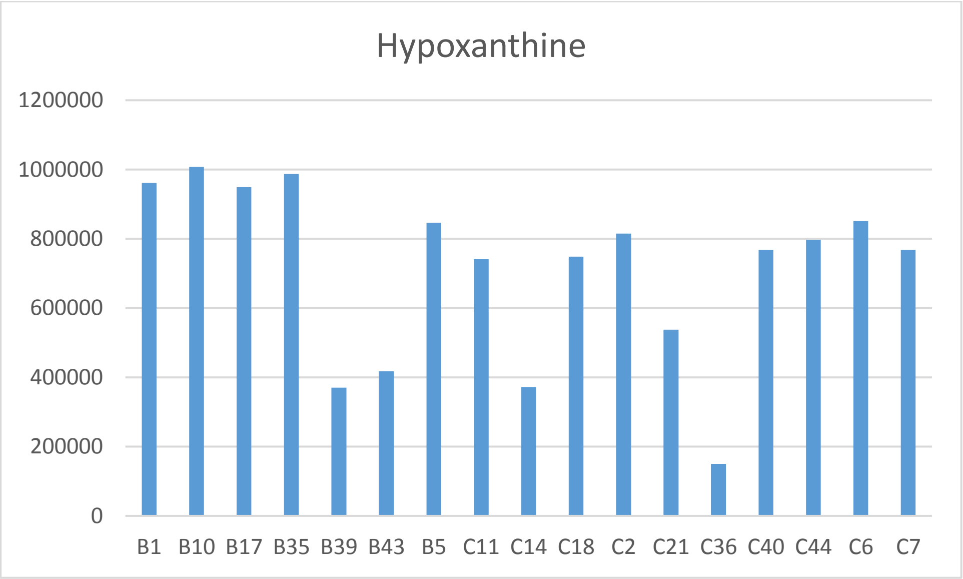

Figure S73 Uridine in pre-80K and post-80K samples

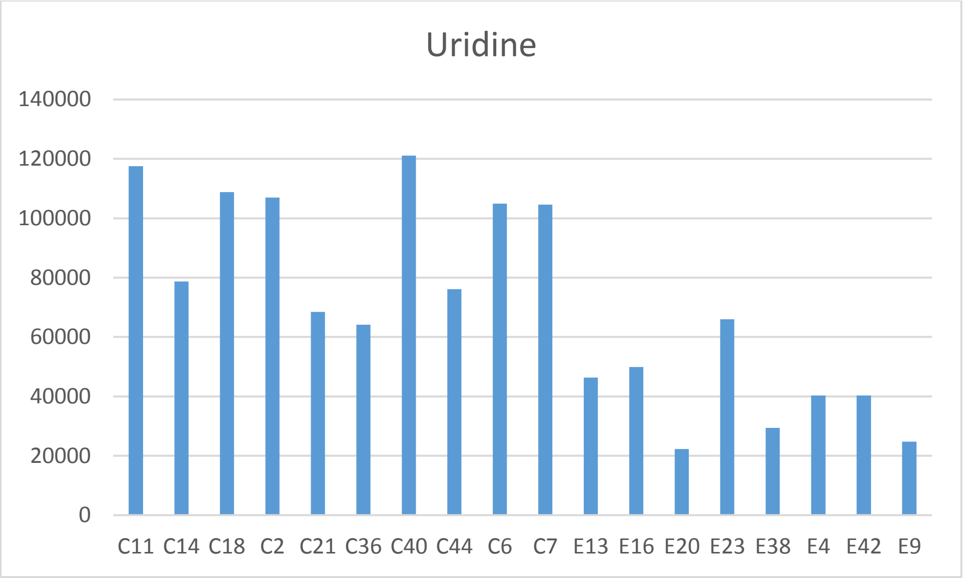

Figure S74 Uridine in baseline and pre-80K samples

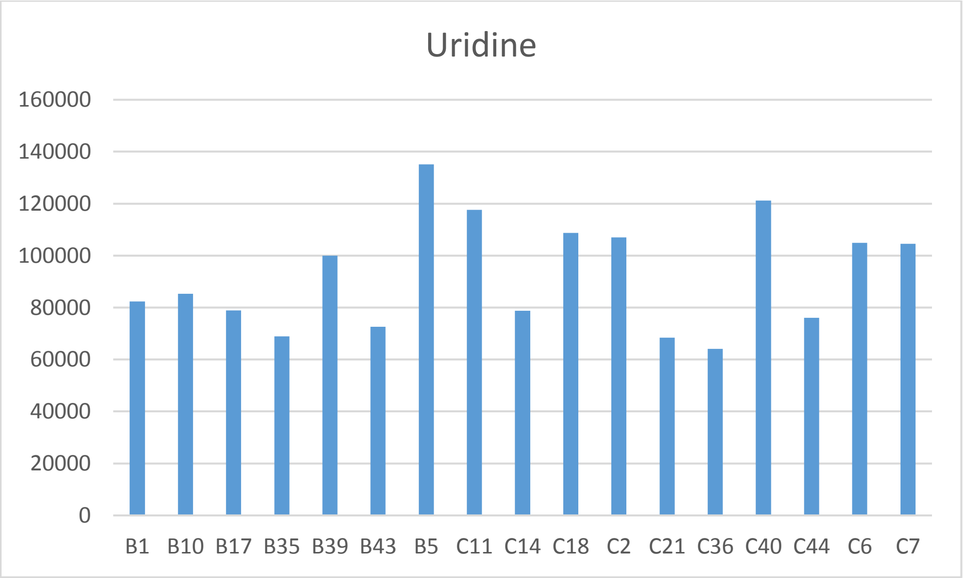

Figure S75 Hydroxycholanate in pre-80K and post-80K samples

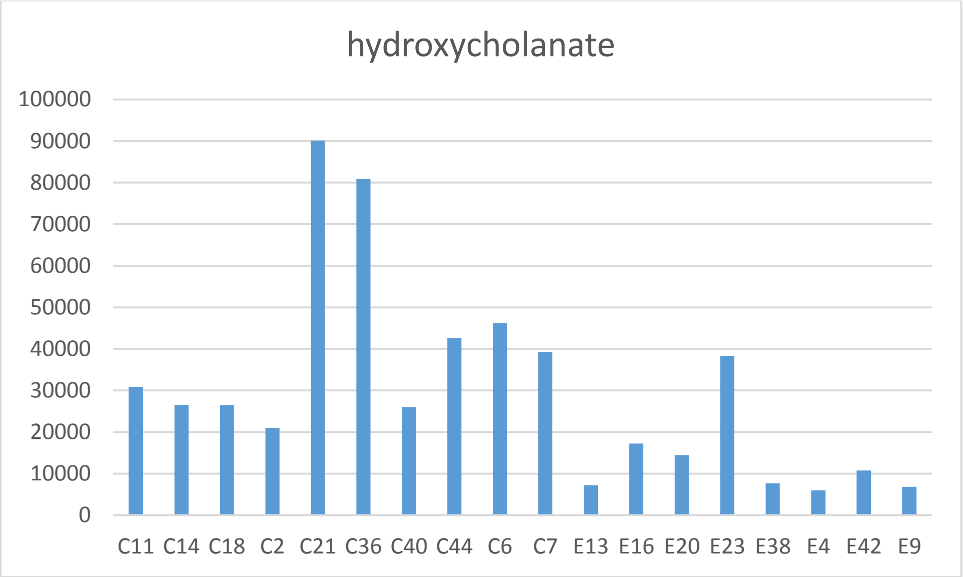

Figure S76 Hydroxycholanate in baseline and pre-80K samples

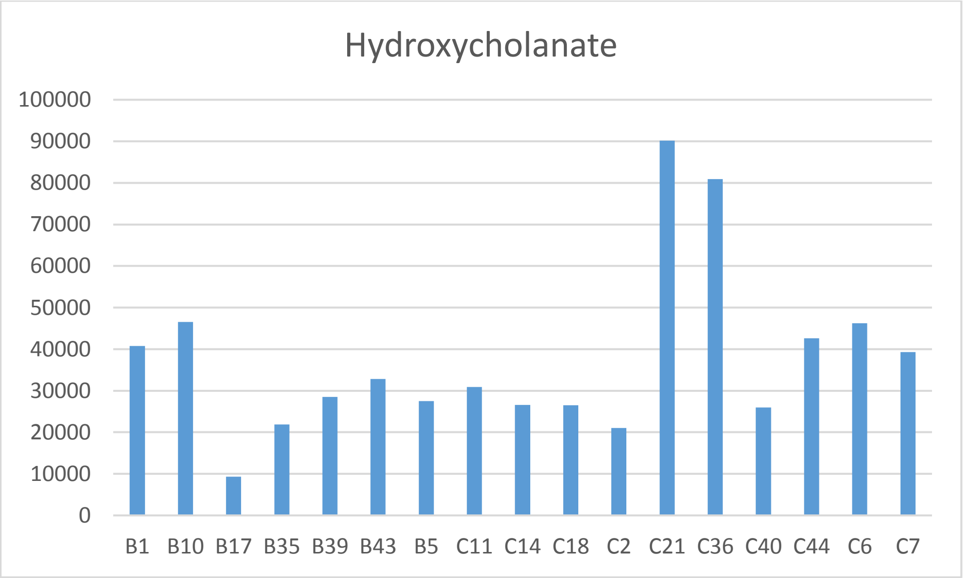

Figure S77 Glycolic acid in pre-80K and post-80K samples

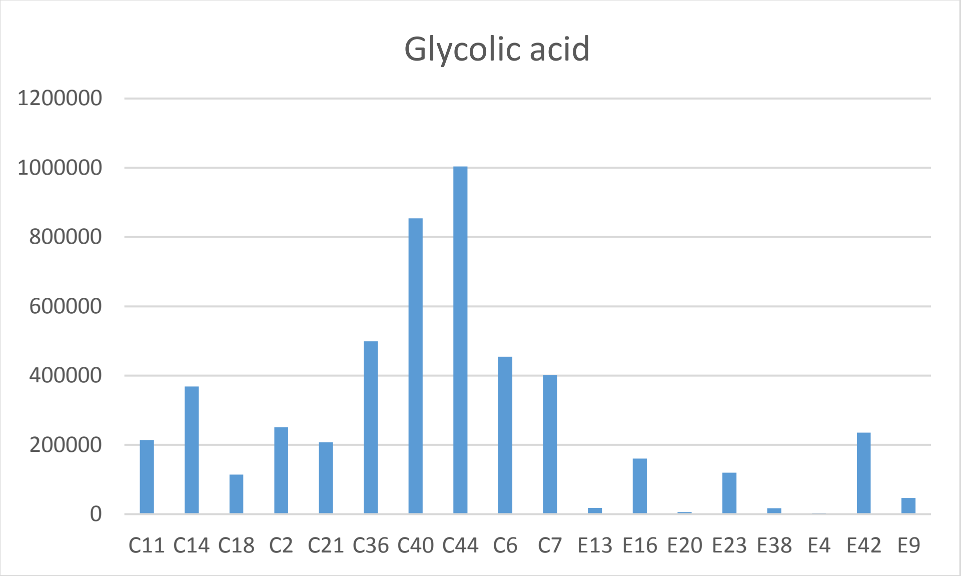

Figure S78 Glycolic acid in baseline and pre-80K samples

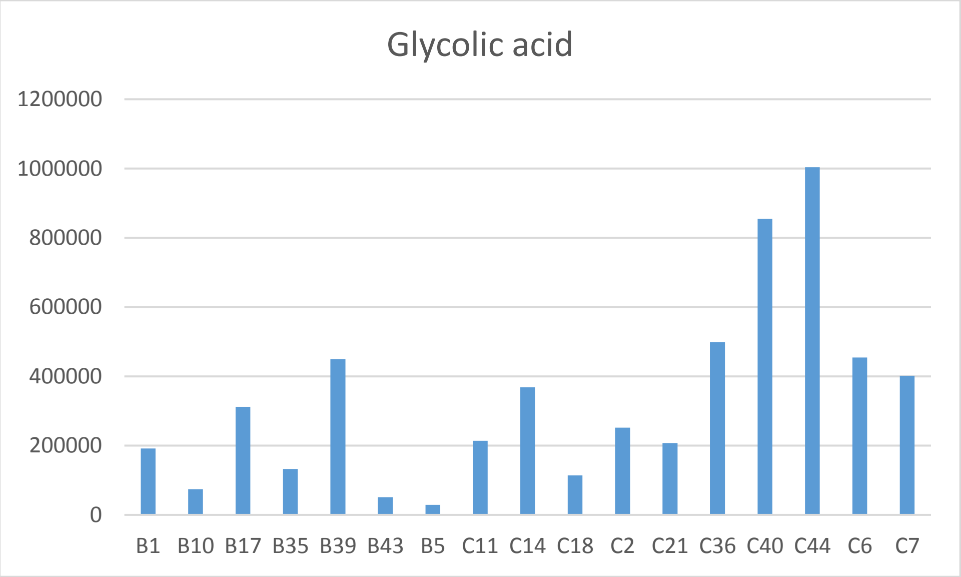

Figure S79 α-tocopherol in pre-80K and post-80K samples

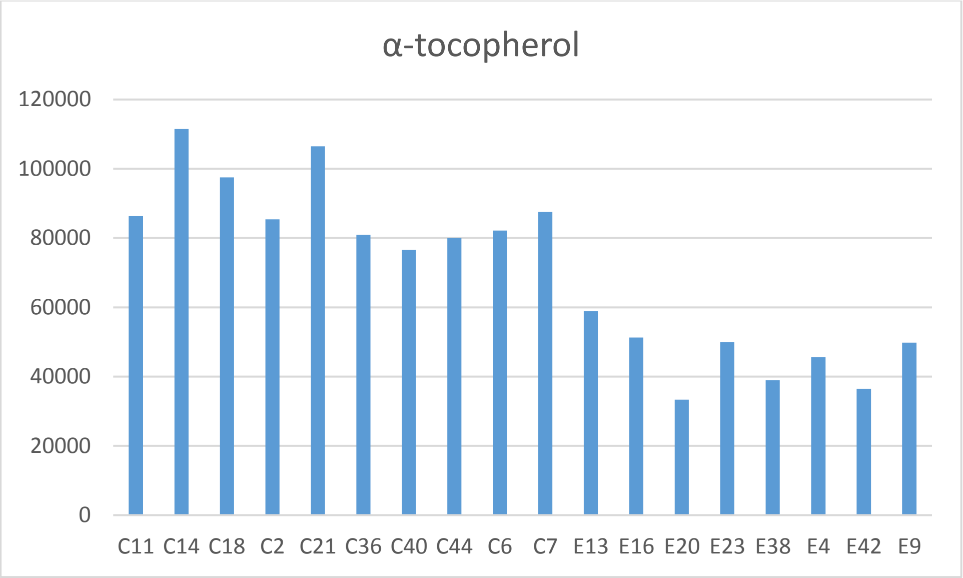

Figure S80 α-tocopherol in baseline and pre-80K samples

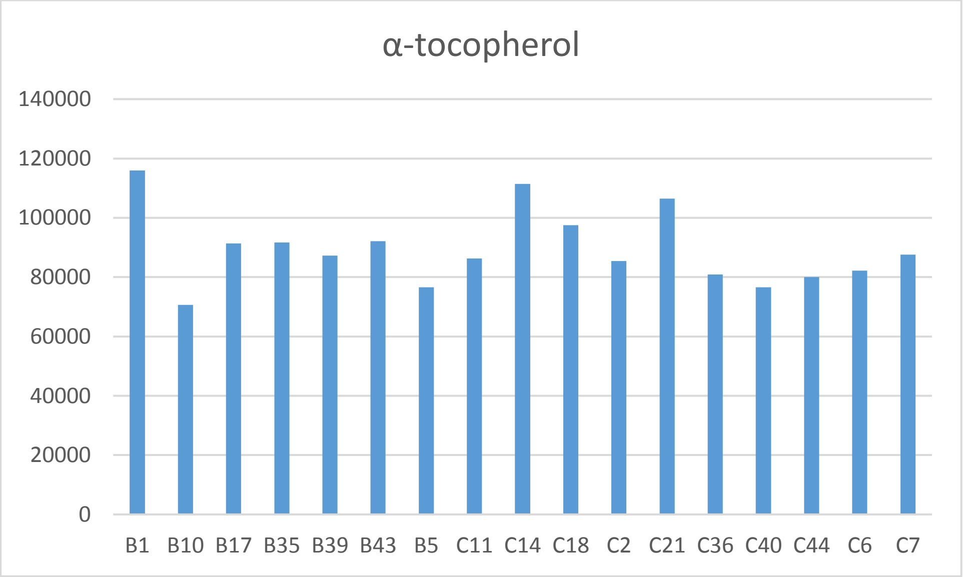

Figure S81 Extracted ion traces for hydroxyoctadecadienoic acids in a pre-80.5km sample and a post-80.5km sample run on an ACE C4 column.

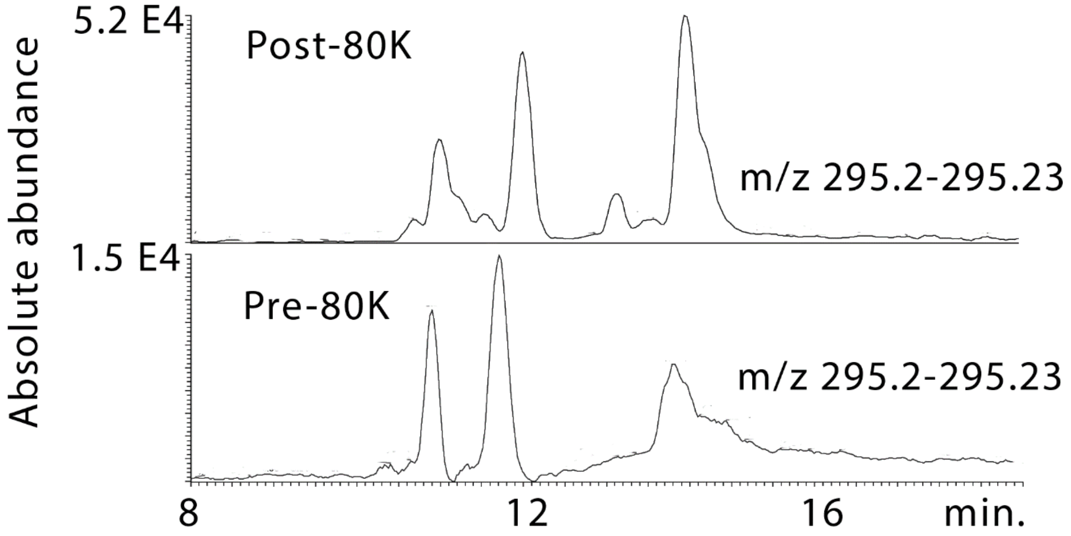

Supplement: Supplementary file 1 [file metabolites-08-00014-s001.pdf]
